# Automated Development of an Accurate Diffusion Database in Fcc AlCoCrFeNi High-Entropy Alloys from a Big Dataset of Composition Profiles

**DOI:** 10.3390/ma15093240

**Published:** 2022-04-30

**Authors:** Jing Zhong, Qin Li, Chunming Deng, Lijun Zhang

**Affiliations:** 1 State Key Laboratory of Powder Metallurgy, Central South University, Changsha 410083, China; zhongjingjogy@csu.edu.cn (J.Z.); qinli333@csu.edu.cn (Q.L.); 2 Institute of New Materials, Guangdong Academy of Sciences, National Engineering Laboratory for Modern Materials Surface Engineering Technology, The Key Lab of Guangdong for Modern Surface Engineering Technology, Guangzhou 510651, China; dengchunming@gdinm.com

**Keywords:** high-entropy alloy, AlCoCrFeNi, diffusion, HitDIC, automation

## Abstract

This study aims to incorporate a big dataset of composition profiles of fcc AlCoCrFeNi alloys, in addition to those of the related subsystem, to develop a self-consistent kinetic description for quinary high-entropy alloys. The latest feature of the HitDIC (**Hi**gh-**t**hroughput **D**etermination of **I**nterdiffusion **C**oefficients) code was adopted in a high-throughput and automatic manner for accommodating a dataset of composition profiles with up to 87 diffusion couples. A good convergence for the optimization process was achieved, while satisfactory results regarding the composition profiles and previously evaluated diffusion properties were obtained. Here, we present an investigation into the elemental effect of Al towards interdiffusion and tracer diffusion, and their potential effect on creep and precipitation processes.

## 1. Introduction

High-entropy alloys (HEAs) represent one of the most appealing research interests in the materials community [1,2,3]. Researchers have developed design strategies to explore the uninvestigated composition space of alloys by combining five and even more equal- /near-equal- atomic components. Among the various kinds of high-entropy alloys, i.e., CoCrFeNi-based HEAs [4,5], Al-TMs HEAs [6] and refractory HEAs [7,8], AlCoCrFeNi HEA are the most widely investigated systems [9,10,11] due to their outstanding mechanical properties and promising heat resistance, making them potential alternatives to conventional high-temperature structural materials, especially for aerospace applications. In systems where precipitation is likely to be introduced, an Al component is employed to match the strength of traditional superalloys at room temperature. Unfortunately, similar to superalloys, the strength and creep performance of such HEAs are prone to degradation at moderate temperatures, i.e., higher than about 800 degrees.

For applications at moderate or high temperatures, creep resistance is an important research priority, especially for traditional superalloy systems [12]. The creep phenomenon mainly takes place when the service temperature of an alloy material is close to its melting point [13]. Meanwhile, the related diffusion rates are rather high, voids are prone to form and cracking tends to evolve at a higher rate, while failure is likely to be encountered for long-time service. Among their different physical properties, the relationship between diffusion coefficients and creep resistance performance has been extensively considered [14,15], though relative investigations have usually been conducted in a qualitative manner without considering the composition or temperature dependence of diffusion coefficients. Moreover, the validity or fundamental cause of sluggish diffusion is still considered a controversial topic among researchers of diffusion and materials [16,17,18], and has not been completely unscrambled due to the absence of a complete overview based on an accurate and well-established diffusion database of HEAs.

In recent years, several contributions have been devoted to investigating the diffusion behaviors of AlCoCrFeNi HEAs [19,20,21,22]. However, the available investigations about the diffusion of AlCoCrFeNi HEAs have been conducted based on a limited dataset of composition profiles, i.e., less than 10 diffusion couples. Furthermore, experimental data related to the sub-system, i.e., CoCrFeNi HEAs, are generally not considered simultaneously. As a result of previous investigation, the most reasonable composition and temperature range of the obtained diffusion descriptions are limited, considering the internal composition and temperature of the employed dataset [23,24]. In cases where the dataset is limited, the assessed diffusion description is at risk of weak extrapolation and interpolation capability from either composition or temperature aspects [23].

In order to obtain reliable diffusion coefficients covering wide composition and temperature regions, the experimental composition profiles of the AlCoCrFeNi alloys, in addition to those of the related subsystem, are considered simultaneously. Despite the large number of composition profiles, the assessment of diffusion descriptions of HEAs is concerned with many descriptors, i.e., binary and ternary interaction parameters of the Relich–Kister polynomial, according to the CALPAHD convention. The assessed results are therefore prone to overfitting and non-uniqueness without a rational strategy for dimensionality reduction and regularization. As the important features of the HitDIC code [24,25,26,27], automation strategies for parameter selection and regularization are implemented, which offer integrated infrastructures for building diffusion descriptions for HEAs in a high-throughput manner.

Consequently, in this study, the diffusion descriptions of the AlCoCrFeNi alloys have been updated using the latest developed parameter selection and regularization strategies [24] to pursue a high quality kinetic description of the desired alloy system. The reliability of the assessed diffusion description is therefore provided, where reliable interdiffusion coefficients and tracer diffusion coefficients are then retained. The influence of Al amount on the diffusion behavior of the fcc AlCoCrFeNi HEA is then inspected with quantified values and straightforward visualization materials. The impact of Al on the related properties concerned with diffusion behavior, i.e., the creep resistance and coarsening rates during precipitation, is then finally carried out for the fcc AlCoCrFeNi HEA.

## 2. Methods

### 2.1. Numerical Inverse Method

To determine the diffusion properties underlying the diffusion phenomenon, numerical inverse methods are adopted, which are feasible for determining the interdiffusion coefficients for systems with any number of components. HitDIC code is developed on the numerical inverse methods and equipped with advanced algorithms for addressing inverse coefficients problem of diffusion. Relatively, the forward problem of diffusion in multi-component systems can be read as

(1) $$\frac{\partial x_{i}}{\partial t}=\nabla \left(\sum_{j=1}^{n-1}{\tilde{D}}_{ij}^{n}\nabla x_{j}\right)$$

where *x_i_* is the composition and ${\tilde{D}}_{ij}^{n}$ is part of the interdiffusion coefficient matrices. For the annealed diffusion couple, the diffusion of components submits to Equation (1), which can be reduced to one dimension along the diffusion direction in a diffusion couple. In Equation (1), the interdiffusion coefficient matrices can be modeled according to Manning’s convention

(2) $${\tilde{D}}_{ij}^{n}=RT\left(M_{i}\Phi_{ij}^{n}-x_{i}\sum_{k}M_{k}\Phi_{kj}^{n}\right)+s\left(M_{i}-\sum_{k}x_{k}M_{k}\right)\frac{2x_{i}RT\sum_{k}M_{k}\Phi_{kj}^{n}}{A_{0}\sum_{k}x_{k}M_{k}}$$

where *R* is the ideal gas constant, *T* the temperature, *M_i_* the atomic mobility and $\phi_{ij}^{n}$ the thermodynamic factor. The second term in Equation (2) is contributed from the vacancy wind effect, where *A*_0_ is the structure factor and s might be assigned with 0 when the attribution is of minimal effect. Furthermore, the atomic mobility can be expanded according to the CALPHAD convention, i.e.,

(3) Φk=∑ixiΦki+∑i∑j>ixixj[∑r=0mrΦki,j(xi−xj)r]+∑i∑j>i∑k>jxixjxk[∑rvrijkΦki,j,kr]+…

From the point view of the numerical inverse method, interdiffusion coefficients can be retrieved by solving the inverse problem related to the diffusion processes during the annealing processes of the diffusion couples. It can be achieved by iteratively adapting the interdiffusion coefficient matrices and simultaneously minimizing the difference between the predictions, based on Equation (1) and observations from diffusion couple experiments. After parametrization, a PDE-constrained optimization problem can be established

(4) $$\min\hat{L}\left(\theta \right)=\min\sum_{i=1}^{N}\sum_{j=1}^{C_{i}}\sum_{k=1}^{n_{j}}{\left\|x_{j,k}^{i}\left(\theta \right)-{\tilde{x}}_{j,k}^{i}\right\|}_{2}^{2}$$

or

(5) $$\min\varepsilon^{2}\left(\theta \right)=\min\frac{1}{N}\sum_{i=1}^{N}\frac{1}{C_{i}}\sum_{j=1}^{C_{i}}\frac{1}{n_{j}}\sum_{k=1}^{n_{j}}{\left\|x_{j,k}^{i}\left(\theta \right)-{\tilde{x}}_{j,k}^{i}\right\|}_{2}^{2}$$

where θ are the parameters of interest, *N* is the number of diffusion couples, *C_i_* denotes the number of components for the *i*-th diffusion couple, *n_j_* is the number of experimental points for the *i*-th diffusion couple, where $x_{j,k}^{i}$ and ${\tilde{x}}_{j,k}^{i}$ are the predicted and observed composition points, respectively. According to the modeling convention of interdiffusion coefficients, the interaction terms in Equation (3), i.e., Φki,jr and Φki,j,kr, can be taken as the parameters of interests, i.e., θ.

### 2.2. Parameter Selection and Regularization Strategy

For dimensionality reduction or parameter selection, the variable selection strategy based on genetic algorithm in HitDIC is employed, where the corrected AIC (Akaike information criterion) is used as the optimization criterion

(6) $$\mathrm{AICc}\left(\theta \right)=2K-2\ln\hat{L}\left(\theta \right)+\frac{2K^{2}+2K}{M-K-1}$$

where *K* is number of activated parameters of interest and *M* is the number of independent observations. *K* is determined according to the auxiliary genes in addition to the genes in the canonical genetic algorithms, which share the same operators in the evolution processes of the genetic algorithm. After parameter selection, the regularization strategy is further introduced to reduce the complexity of the diffusion models, i.e., the L2 norm of the parameters of interest. The regularization process starts from the optimal output from the parameter selection process, while the regularization term λ is subsequently screened from a very small number, i.e., 10^−9^. During the regularization process, the penalized target is adopted as the optimization criterion

(7) $$F=\hat{L}+\lambda {\left\|\theta \right\|}_{2}^{2}$$

## 3. Results and Discussion

### 3.1. Diffusion Database in Fcc AlCoCrFeNi HEAs

A re-evaluation study case was carried out, where the composition profiles of fcc AlCoCrFeNi alloy and related quaternary sub-system were joined, as listed in Table 1. A big dataset with a total of 87 composition profiles were therefore built. Subsequently, a pre-processing process, i.e., denoising experimental data, was also applied before the workflow of parameter selection and regularization processes.

**Figure 1:**
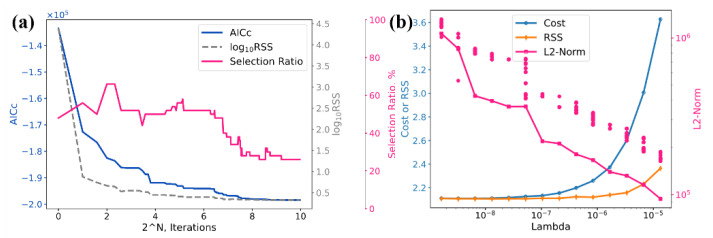
Convergence sequence of the parameter selection strategy and the regularization strategy: (**a**) Parameter selection strategy; (**b**) Regularization strategy.

In addition to the expanded experimental dataset, advanced algorithms, i.e., parameter-selection strategy and regularization strategy, were applied for rigorous assessment over the related interaction parameters of the kinetic database. The convergence sequence of the parameter selection strategy is shown in Figure 1a, where an overall selection ratio of about 26% is achieved. During optimization, all the endmembers were adopted from Li et al., while the zero order of binary interaction parameters were assigned as unknowns to be optimized according to the AICc (corrected Akaike information criterion). The selected parameters, i.e., 13 out of 50 parameters, were therefore employed using the regularization strategy, as shown Figure 1b. During regularization, the cost, i.e., the penalized residual summation of square, was adopted as the optimization target with a different level of penalty, i.e., the regularization term or Lambda. L2 norm stands for the model complexity, and it is attributed to the overall cost by multiplying the regularization term. For the sake of goodness of fit and generalization, the regularization of $10^{-6}$ was chosen as the reasonable level.

**Figure 2:**
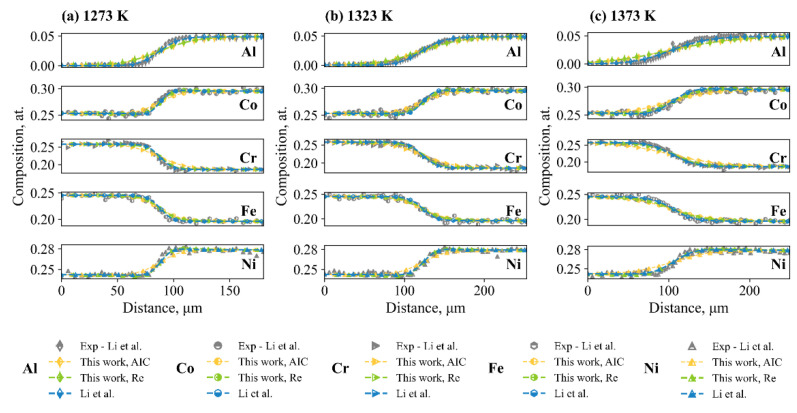
Comparison of the typical composition profiles among the experimental results (Li et al. [20]) and model-predicted results due to Li et al., optimization with parameter selection based on AICc and optimization with regularization based on L2 norm penalty: (**a**) 1273 K; (**b**) 1323 K; (**c**) 1373 K.

**Figure 3:**
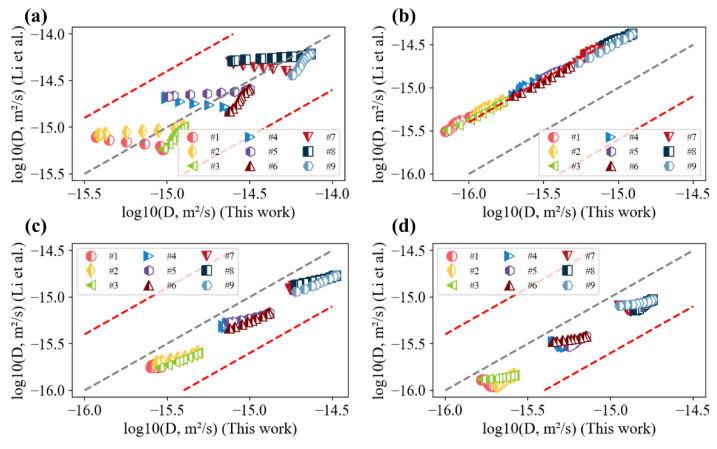
Comparison between the interdiffusion coefficients of different diffusion couples (noted with #1–#3 for 1273 K and 48 h, #4–#6 for 1323 K and 48 h, and #7–#9 for 1373 K and 48 h, evaluated by Li et al. [20] as listed in Table 1) and optimization results in the present work: (**a**) ${\tilde{D}}_{\mathrm{AlAl}}^{\mathrm{Ni}}$; (**b**) ${\tilde{D}}_{\mathrm{CoCo}}^{\mathrm{Ni}}$; (**c**) ${\tilde{D}}_{\mathrm{CrCr}}^{\mathrm{Ni}}$; (**d**) ${\tilde{D}}_{\mathrm{FeFe}}^{\mathrm{Ni}}$.

To be clear, goodness of fit was observed between the model-predicted results and experimental composition profiles, as shown in the Supplementary File. Specifically, all optimization processes by the present work and Li et al. [20] were able to generate satisfactory predictions results towards the experimental composition profiles, as shown in Figure 2. The evaluated interdiffusion coefficients were therefore compared with the results by Li et al., as shown in Figure 3. Reasonable agreement was found between the interdiffusion coefficients using the two datasets, i.e., the one from Li et al. and the ones with additional composition profiles for fcc CoCrFeNi systems. Differences in the obtained interdiffusion coefficients between Li et al. and the present work are less than half a magnitude among the composition space of experimental composition profiles, which is considered reasonable.

**Figure 4:**
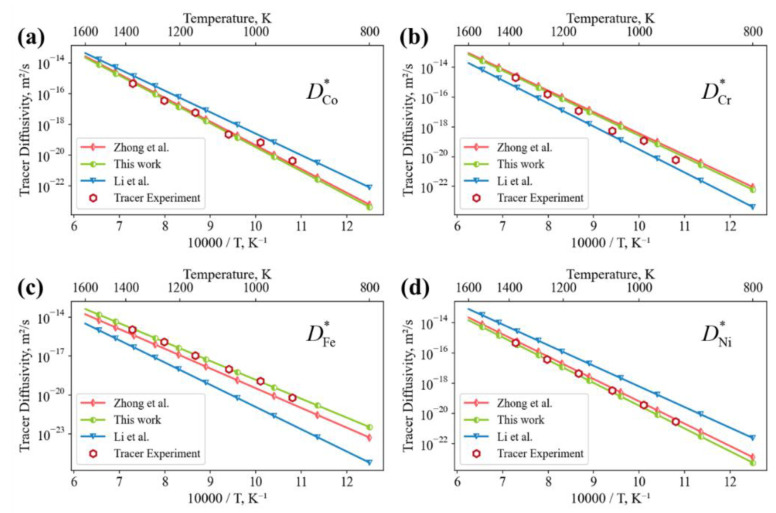
Comparison between the tracer diffusion coefficients evaluated by different researchers [20,24,35] and optimization results in the present work: (**a**) $D_{\mathrm{Co}}^{*}$; (**b**) $D_{\mathrm{Cr}}^{*}$; (**c**) $D_{\mathrm{Fe}}^{*}$; (**d**) $D_{\mathrm{Ni}}^{*}$.

For equal-atomic high-entropy alloys, the ideal mixing theory was applied and the related tracer diffusion coefficients towards the quinary systems were retrieved, as shown in Figure 4. Reasonable agreement is found among evaluated tracer diffusion coefficients of the fcc CoCrFeNi alloy by this work, Li et al. [20] and tracer experiments [35]. Those by Li et al. merely consider composition profiles of the quinary systems, however the interpolation performance toward the quaternary system reamins satisfactory. Considering our previous evaluated description based on the dataset of the fcc CoCrFeMnNi alloy and related sub-systems [24], a minor difference is observed against the evaluated results. The results of diffusion description evaluated from the dataset of a large number of composition profiles indicate that they share a common essence among the overlapped composition space for the concerned system.

### 3.2. Effect of Al Amount on Diffusion, Creep Resistance and Precipitation Behaviors of AlCoCrFeNi HEAs

Addition of Al is an important strategy for achieving lightweight high-entropy alloys, while precipitations are likely introduced due to their promising mechanical performance. However, complex thermodynamic, kinetic and mechanical effects might emerge with varying amounts of Al [9,10,11]. For high-entropy alloys, the related high temperature performance is currently deemed as limited, which is generally due to its diffusion properties at ambient temperatures. Therefore, an investigation into diffusion properties is essential for identifying the most appropriate amount of Al.

**Figure 5:**
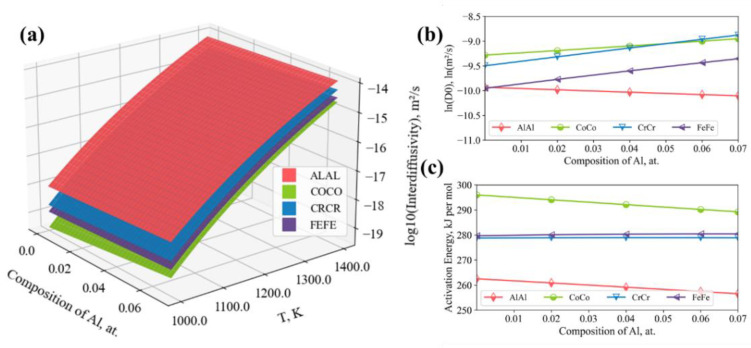
Influence of aluminum content on the main terms of the interdiffusivity matrices of the Alx(CoCrFeNi)1-x alloys: (**a**) Surface plot for interdiffusivites; (**b**) Pre-frequency factors of Arrhenius relation; (**c**) Activation energies of the Arrhenius relation, where ALAL stands for ${\tilde{D}}_{\mathrm{AlAl}}^{\mathrm{Ni}}$, COCO for ${\tilde{D}}_{\mathrm{CoCo}}^{\mathrm{Ni}}$, CRCR for ${\tilde{D}}_{\mathrm{CrCr}}^{\mathrm{Ni}}$ and FEFE for ${\tilde{D}}_{\mathrm{FeFe}}^{\mathrm{Ni}}$.

With the assessed diffusion description for the fcc AlCoCrFeNi alloy in the present work, the surface of the main terms of the interdiffusion coefficient matrices are shown in Figure 5a. Comparing the main terms at the same temperature, it was observed that ${\tilde{D}}_{\mathrm{AlAl}}^{\mathrm{Ni}}$ > ${\tilde{D}}_{\mathrm{CrCr}}^{\mathrm{Ni}}$ > ${\tilde{D}}_{\mathrm{FeFe}}^{\mathrm{Ni}}$ > ${\tilde{D}}_{\mathrm{CoCo}}^{\mathrm{Ni}}$. Moreover, the diffusion coefficients are under the principle of the Arrhenius relation and the related parameters for the Arrhenius relation are presented in Figure 5b,c. For the pre-frequency factor, the amount of Al mainly contributes to the increment of $D_{\mathrm{CoCo}}^{0}$, $D_{\mathrm{CrCr}}^{0}$ and $D_{\mathrm{FeFe}}^{0}$, leading to a downward trend for $D_{\mathrm{AlAl}}^{0}$. In terms of the activation energies, $Q_{\mathrm{AlAl}}$ and $Q_{\mathrm{CoCo}}$ decrease as the amount of Al increases, while no significant influence on $Q_{\mathrm{CrCr}}$ and $Q_{\mathrm{FeFe}}$ was observed.

**Figure 6:**
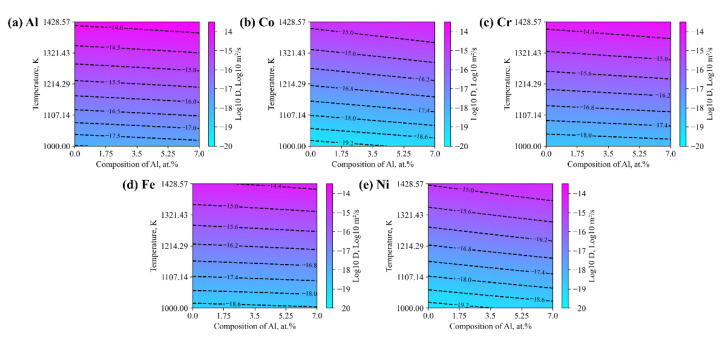
Influence of aluminum content on the tracer diffusion coefficients of the Alx(CoCrFeNi)1-x alloys: (**a**) $D_{\mathrm{Al}}^{*}$; (**b**) $D_{\mathrm{Co}}^{*}$; (**c**) $D_{\mathrm{Cr}}^{*}$; (**d**) $D_{\mathrm{Fe}}^{*}$; (**e**) $D_{\mathrm{Ni}}^{*}$.

**Figure 7:**
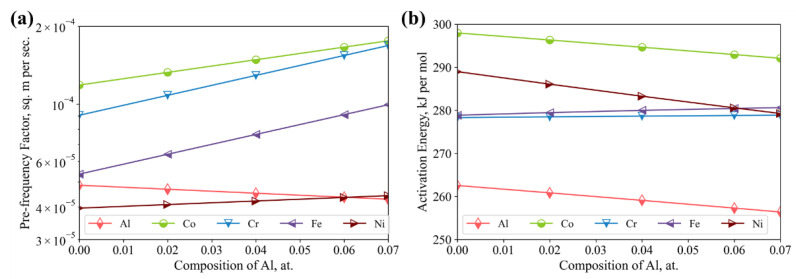
Influence of aluminum content on (**a**) pre-frequency factors and (**b**) activation energies of the tracer diffusion coefficients of the Alx(CoCrFeNi)1−x alloys.

In addition to the interdiffusivities, the corresponding tracer diffusion coefficients for the Al_x_(CoCrFeNi)_1−x_ is calculated as shown in Figure 6. Among the tracer diffusion coefficients shown in Figure 6a, the diffusion rate increased as the amount of Al content increased. A slightly stronger enhancing effect on the tracer diffusion coefficients, i.e., $D_{\mathrm{Co}}^{*}$ and $D_{\mathrm{Ni}}^{*}$, is observed, though the related tracer diffusion coefficients for Co and Ni are almost lower than the others by a half of a magnitude, with respect to the same temperature. Overall, the diffusion rates of Al are dominantly faster than the others, especially in regions with a higher temperature. A closer look can be taken by reducing the tracer diffusion coefficients to the related Arrhenius relation, i.e., the pre-frequency factor and activation energy, as shown in Figure 7. In terms of the pre-frequency factor, those of $D_{\mathrm{Al}}^{*}$ and $D_{\mathrm{Ni}}^{*}$ are less sensitive to the amount of Al, while having a significant effect on the those of $D_{\mathrm{Co}}^{*}$, $D_{\mathrm{Cr}}^{*}$ and $D_{\mathrm{Fe}}^{*}$, which increase as the amount of Al increase. The ordering sequence of the pre-frequency factors read as $D_{\mathrm{Co}}^{0}$ > $D_{\mathrm{Cr}}^{0}$ > $D_{\mathrm{Fe}}^{0}$ > $D_{\mathrm{Al}}^{0}$ > $D_{\mathrm{Ni}}^{0}$, where those of $D_{\mathrm{Co}}^{*}$ and $D_{\mathrm{Cr}}^{*}$ are dominantly larger than the others, considering the same amount of Al. Similarly, the ranking of the activation energies of the corresponding coefficients is $Q_{\mathrm{Co}}$ > $Q_{\mathrm{Ni}}$ > $Q_{\mathrm{Fe}}$ > $Q_{\mathrm{Cr}}$ > $Q_{\mathrm{Al}}$. Such a tendency coincides well with order of atom mass of the corresponding components, i.e., ${\mathrm{Ar}}_{\mathrm{Co}}$ > ${\mathrm{Ar}}_{\mathrm{Ni}}$ > ${\mathrm{Ar}}_{\mathrm{Fe}}$ > ${\mathrm{Ar}}_{\mathrm{Cr}}$ > ${\mathrm{Ar}}_{\mathrm{Al}}$. It is therefore inferred that the assessed diffusion properties not only restore the underlying physics of the components but also reasonably address the intricate interactions among the components.

For HEAs, the diffusion coefficient is an important transport coefficient for describing many diffusion-controlled processes, i.e., solidification, aging and creep processes at a moderate service temperature. For solidification, the diffusion rates are attributed to the subtle micro-segregation of the alloying components in the concerned system. Generally, numerical simulation techniques, i.e., Scheil solidification simulation and phase-field modeling, are able to provide a good representation of the state in a material during solidification, which usually occurs in non-equilibrium conditions. Such application scenarios of the diffusion database resemble the demands of other preparation or service processes, where the quantitative description of mass transportation is indispensable.

In addition to the above computation intensive simulation techniques, efforts have also been paid to the development of empirical or semi-empirical models for evaluating the performance indexes of the corresponding processes, i.e., creep processes. Zhu et al. [36] introduces an analytic model for the prediction of the shear strain rate for superalloys during the creep period, where the effective diffusion coefficients are considered to contribute directly to such a quantity. Reed et al. further proposes a merit index according to effective diffusion coefficients, which is calculated by summating the reciprocal diffusion coefficients weighted by the corresponding compositions [15]. Liu et al. [14] carries out modeling of creep merit indexes according to the Reed’s model, where the tracer diffusion coefficients are employed alternatively, i.e.,

(8) $$M_{\mathrm{creep}}=\sum_{i}\frac{x_{i}}{D_{i}^{*}}$$

**Figure 8:**
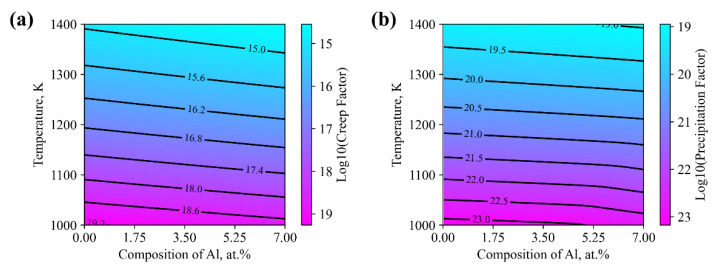
Evaluated (**a**) creep factor and (**b**) precipitation factor of the Alx(CoCrFeNi)1-x alloys based on the assessed kinetic description.

Following the above convention, the evaluated creep merit index is calculated and illustrated in Figure 8a. Among the concerned temperature range, it is found that the creep merit index decreases significantly. The tendency of composition dependence is also observed, where the increment of Al content led to the degradation of the creep merit index. It is thus indicated that the amount of Al is significant in controlling the high temperature performance of AlCoCrFeNi HEAs.

To efficiently employ the principle of the assessed description, a precipitation factor is proposed, referring to the convention of the creep merit index. For the coarsening process during the precipitation period, Morral and Purdy [37] proposes the particle coarsening formula for a multi-component system, where inversion of the chemical mobility matrices, i.e., ${\left[M\right]}^{-1}$, is the diffusion factor presented in the expression of the coarsening rate coefficient. To illustrate the effect of Al, the chemical mobility matrices are evaluated, while inversion of the maximum characteristic value of the corresponding matrices is calculated, as shown in Figure 8b. According to the coarsening formula, it is indicated that the larger the precipitation factor, the slower the coarsening rate. Similar to the creep merit index, the temperature dependence of the precipitation factor is significant, which drops with the increase in temperature. It is also found that the increment of the amount of Al contributes to the reduction of the corresponding precipitation factor, where the rate of coarsening is deemed to be increased in principle, though the dependence on composition is considered less significant compared with the creep merit index.

As a brief summary, the assessed diffusion database of AlCoCrFeNi alloys can successfully capture the composition and temperature dependency for kinetic essence. That is, the deduced properties, i.e., interdiffusion coefficients and tracer diffusion coefficients, are consistent with the reported calculation or experimental results. Moreover, the inferred quantities, i.e., the creep merit index and precipitation factor, also demonstrate a common tendency to influence the alloying component, i.e., Al, though the relevancy of this argument is yet to be further determined. Such a priority is considered to originate from the adopted strategy of parameter selection and regularization, which can efficiently facilitate the big dataset of diffusion information.

## 4. Conclusions

- The diffusion description of the fcc AlCoCrFeNi alloy is automatically evaluated using the latest version of HitDIC software based on a big dataset of composition profiles, composed of fcc AlCoCrFeNi HEA and the related subsystem. Good convergence behaviors are achieved for the automation strategies of parameter selection and regularization, while the obtained diffusion description can reasonably reproduce all composition profiles in the dataset related to the fcc AlCoCrFeNi HEAs.
- Model-predicted interdiffusion and tracer diffusion coefficients shows good consistency with the reported interdiffusion coefficients and tracer diffusion coefficients, respectively. Generality and accuracy of the employed parameter selection and regularization strategies are therefore proved, as well as the robustness of the HitDIC code.
- Related diffusion properties, including the interdiffusion coefficients, tracer diffusion coefficients, creep index and the precipitation factor, are subsequently evaluated. Addition of Al tends to provide lower activation energies for Al, Co and Ni components, while no significant contribution is noted towards Cr and Fe, especially for tracer diffusion. Addition of Al is found to result in the decline in creep resistance, while the related coarsening rate tends to increase with the increment of Al amount.

## Figures and Tables

**Table 1:** List of the employed diffusion couple experimental information (The total number of diffusion couples subject to “Information” column is listed in “Number” column with specific counts in the brackets for different annealing temperature or time).

Systems	Information	Number	Ref.
fcc AlCoCrFeNi	1273 K, 100 h; 1323 K, 75 h; 137 3K, 50 h;	8(3/2/3)	[19]
	1273 K, 48 h; 1323 K, 48 h; 137 3K, 48 h;	9(3/3/3)	[20]
	1173 K, 240 h; 1273 K, 120 h; 1373 K, 48 h; 1473 K, 24 h;	4(1/1/1/1)	[22]
fcc CoCrFeNi	1273 K, 50 h;	1	[28]
	1423 K, 100 h; 1423 K, 240 h	2(1/1)	[29]
	1343 K, 94.5 h;	1	[30]
	1350 K, 72 h;	8	[31]
	1273 K, 100 h;	3	[32]
	1350 K, 72 h; 1310 K, 72 h; 1270 K, 96 h; 1230 K, 96 h;	40(10/10/10/10)	[33]
	1173 K, 100 h; 1273 K, 100 h; 1355 K, 100 h; 1198 K, 100 h; 1198 K, 400 h; 1198 K, 900 h;	11(6/1/1/1/1/1)	[34]

## Data Availability

The relevant data and scripts used in the study are available when requested.

## Supplementary Materials

The following supporting information can be downloaded at: https://www.mdpi.com/article/10.3390/ma15093240/s1, Figures S1–S87. Refs. [19,20,22,28,29,30,31,32,33,34].
