# Supplementary material for: Automated Development of an Accurate Diffusion Database in Fcc AlCoCrFeNi High-Entropy Alloys from a Big Dataset of Composition Profiles
Source: Materials (Basel). 2022 Apr 30;15(9):3240. doi: 10.3390/ma15093240 (PMC9102832; doi:10.3390/ma15093240)
Supplement: Supplementary file 1 [file materials-15-03240-s001.zip › materials-1645802-supplementary.pdf]

Supplementary File
Comparison between the modeled-predicted and experimental composition profiles

Jing Zhong, Qin Li, Chunming Deng, Lijun Zhang*
*lijun.zhang@csu.edu.cn;

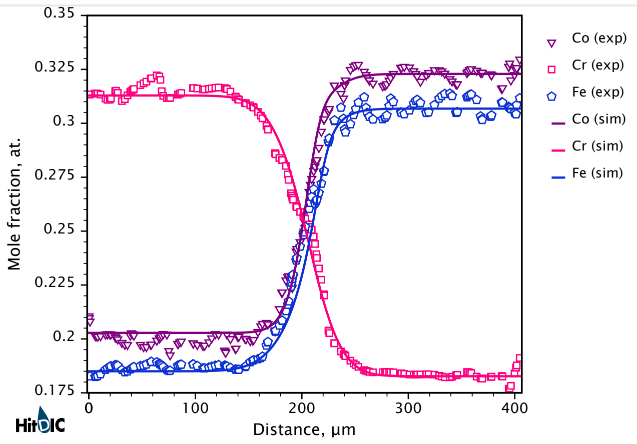

Figure S1 Dabrowa1019-fig10a

1350 K / 259200 sec / 0.00020416619372554123 m

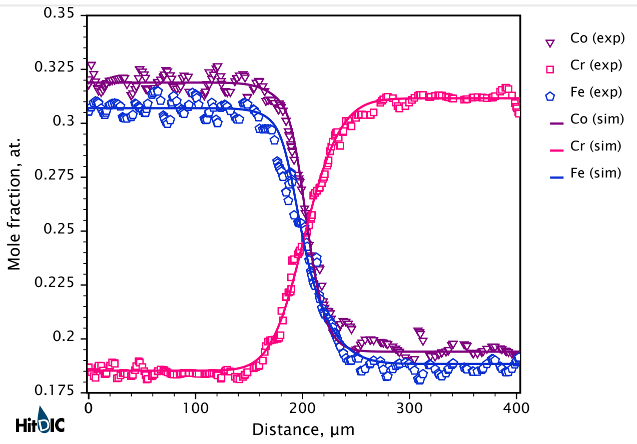

Figure S2 Dabrowa1019-fig10b

1350 K / 259200 sec / 0.00020321269403211772 m

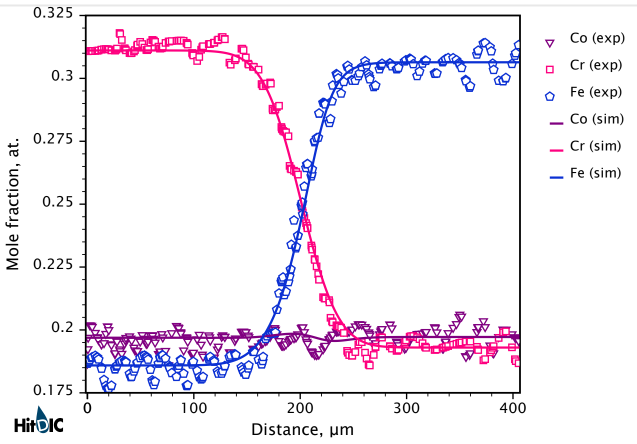

Figure S3 Dabrowa1019-fig10c

1350 K / 259200 sec / 0.0002001449029194191 m

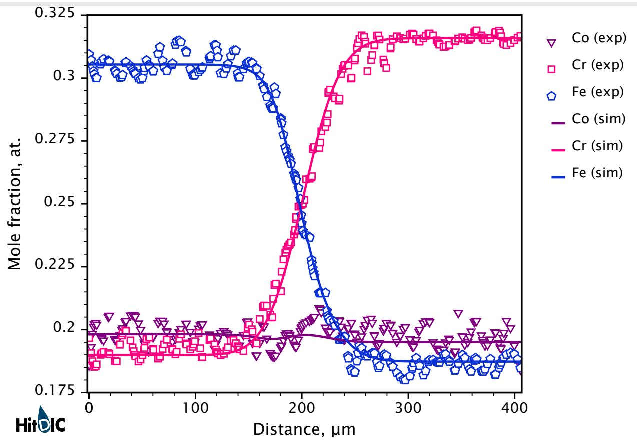

Figure S4 Dabrowa1019-fig10d

1350 K / 259200 sec / 0.0002014937053900212 m

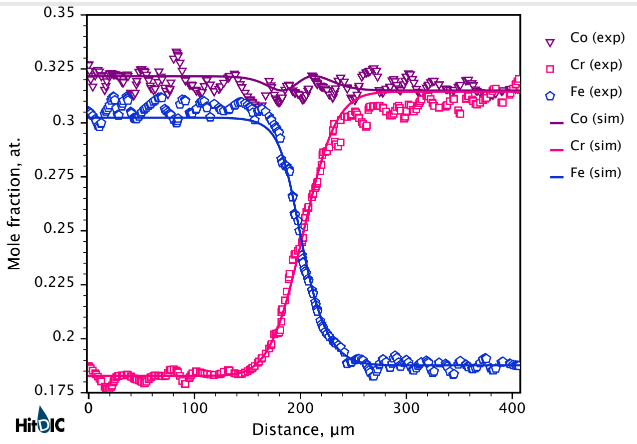

Figure S5 Dabrowa1019-fig10e

1350 K / 259200 sec / 0.00020263080659788102 m

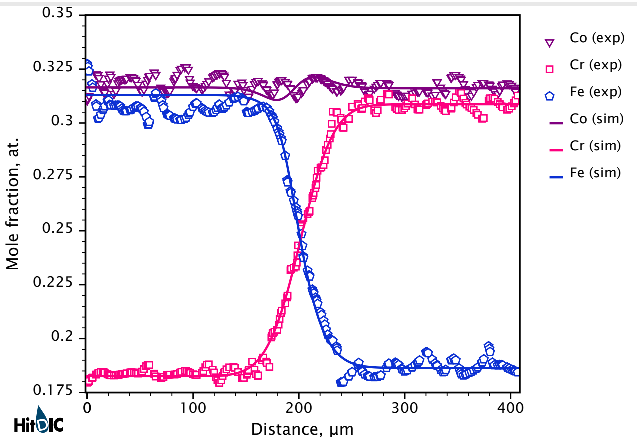

Figure S6 Dabrowa1019-fig10f

1350 K / 259200 sec / 0.00020142599532846361 m

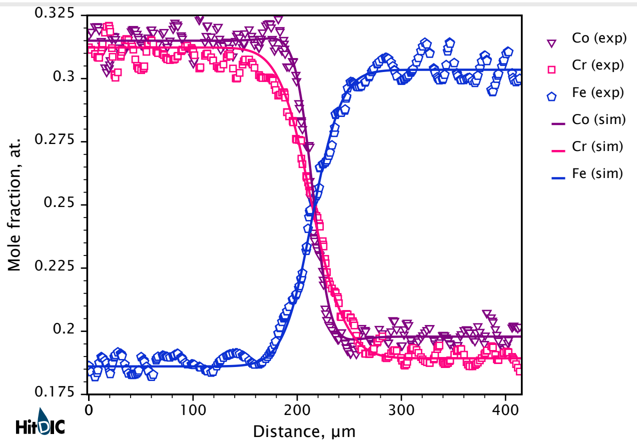

Figure S7 Dabrowa1019-fig10g

1350 K / 259200 sec / 0.00021447709877975285 m

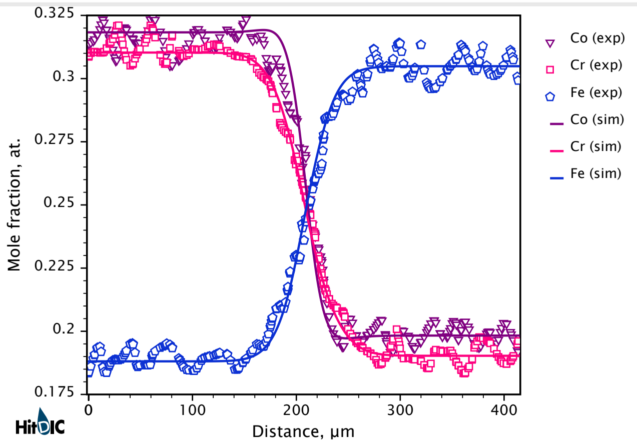

Figure S8 Dabrowa1019-fig10h

1350 K / 259200 sec / 0.00020866779959760606 m

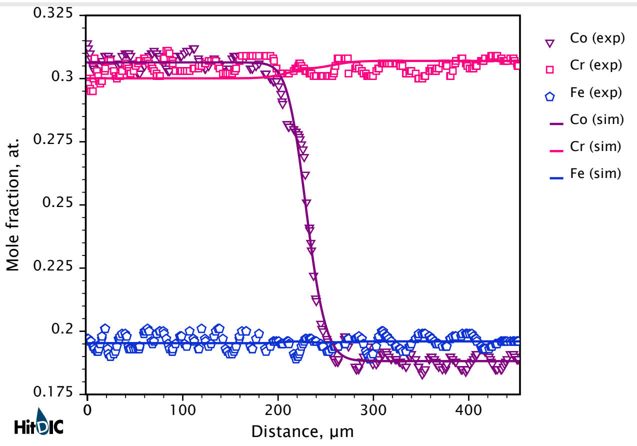

Figure S9 Dabrowa1019-fig11a

1350 K / 259200 sec / 0.0002303041983395815 m

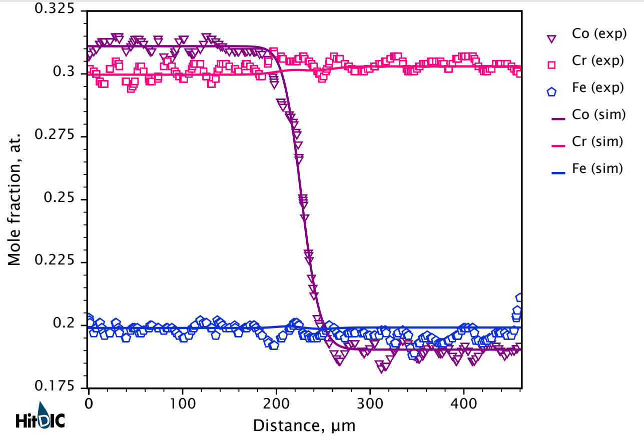

Figure S10 Dabrowa1019-fig11b

1350 K / 259200 sec / 0.00022739899577572942 m

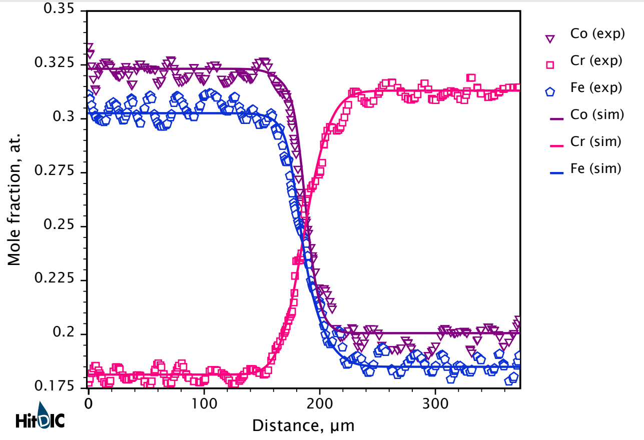

Figure S11 Dabrowa1019-fig11c

1310 K / 259200 sec / 0.00018705629918258637 m

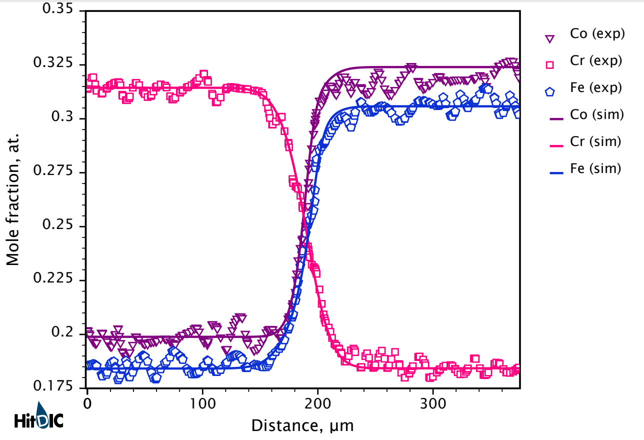

Figure S12 Dabrowa1019-fig11d

1310 K / 259200 sec / 0.0001890654966700822 m

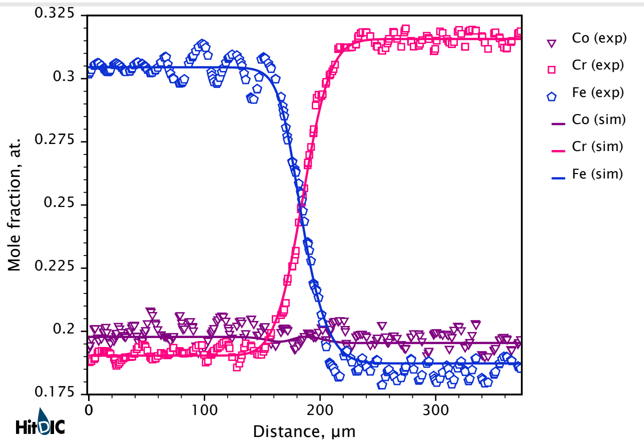

Figure S13 Dabrowa1019-fig11e

1310 K / 259200 sec / 0.00018522399477660656 m

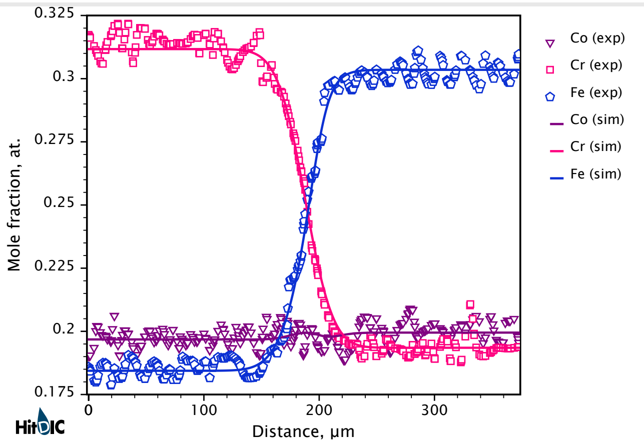

Figure S14 Dabrowa1019-fig11f

1310 K / 259200 sec / 0.00018754010670818388 m

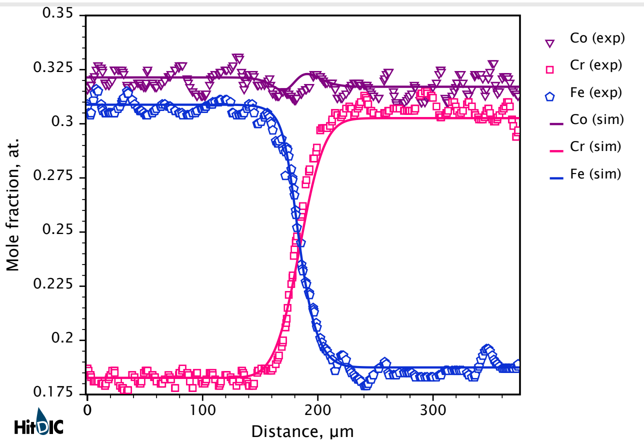

Figure S15 Dabrowa1019-fig11g

1310 K / 259200 sec / 0.0001840918994275853 m

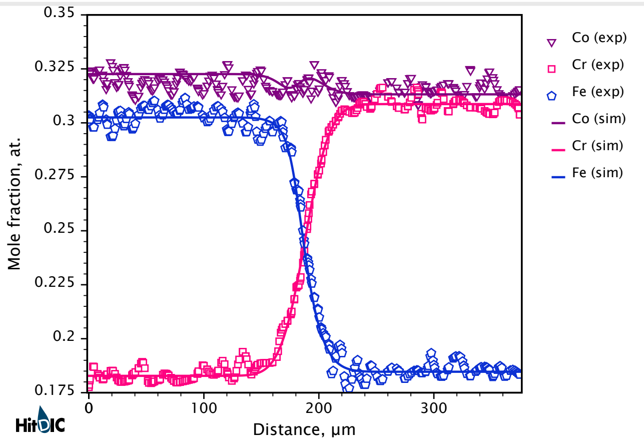

Figure S16 Dabrowa1019-fig11h

1310 K / 259200 sec / 0.00018787100270856172 m

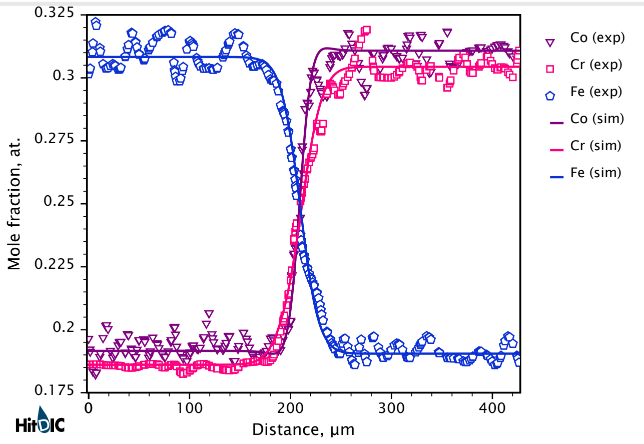

Figure S17 Dabrowa1019-fig12a

1310 K / 259200 sec / 0.0002092081995215267 m

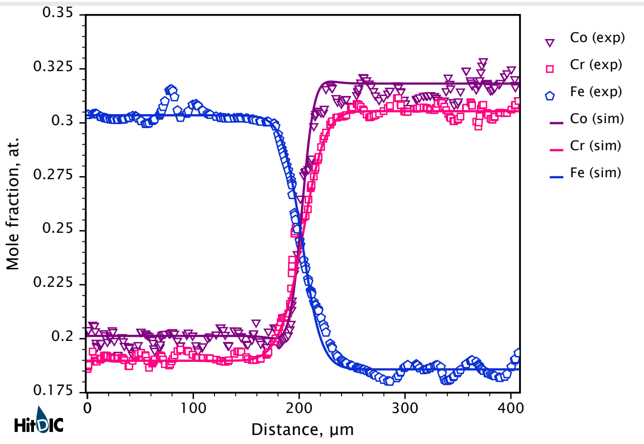

Figure S18 Dabrowa1019-fig12b

1310 K / 259200 sec / 0.00020272140682209283 m

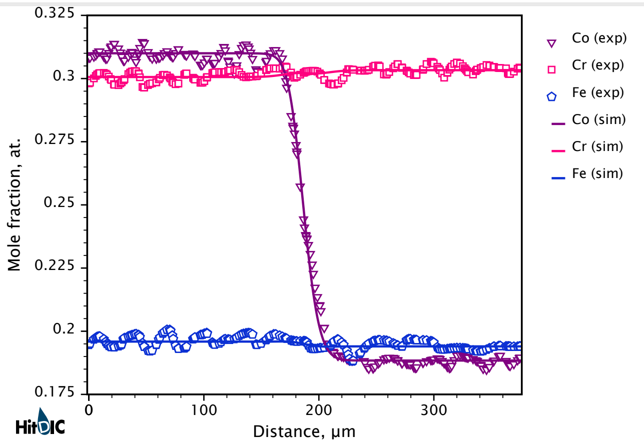

Figure S19 Dabrowa1019-fig12c

1310 K / 259200 sec / 0.0001868565013865009 m

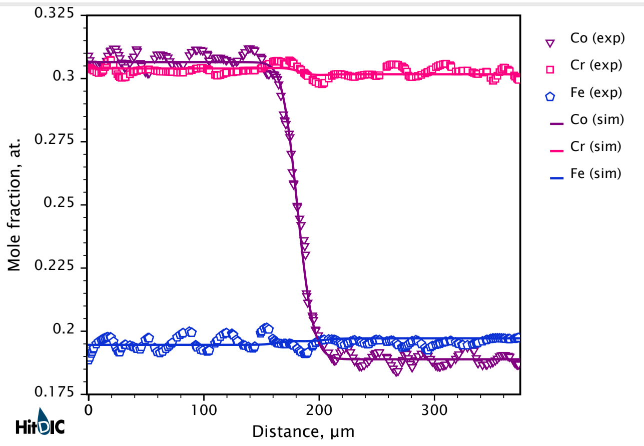

Figure S20 Dabrowa1019-fig12d

1310 K / 259200 sec / 0.00018162300693802536 m

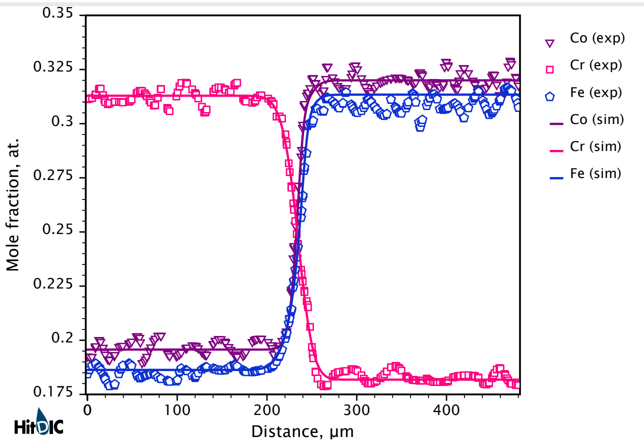

Figure S21 Dabrowa1019-fig12e

1270 K / 345600 sec / 0.00023406679974868894 m

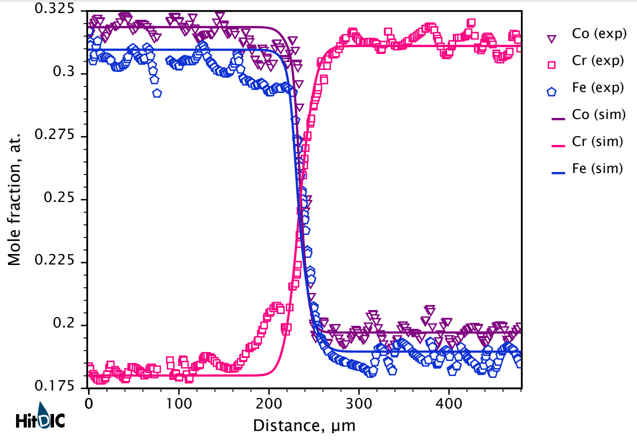

Figure S22 Dabrowa1019-fig12f

1270 K / 345600 sec / 0.00023415310715790838 m

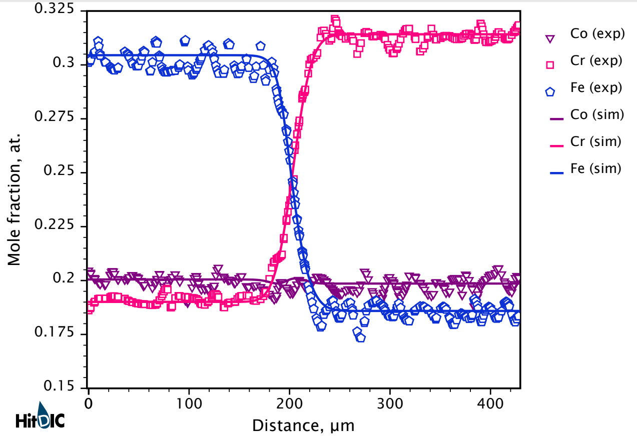

Figure S23 Dabrowa1019-fig12g

1270 K / 345600 sec / 0.00020367279648780823 m

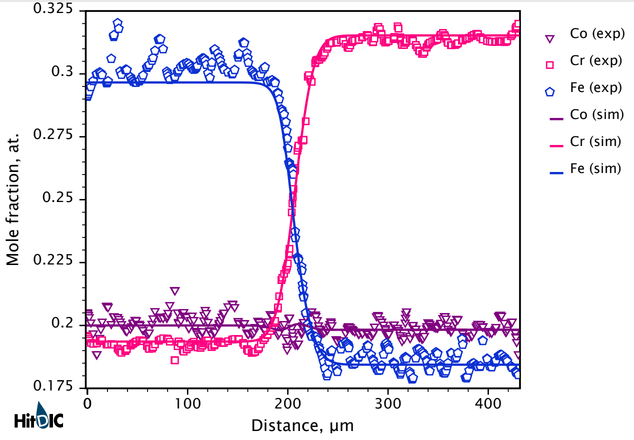

Figure S24 Dabrowa1019-fig12h

1270 K / 345600 sec / 0.0002074093063129112 m

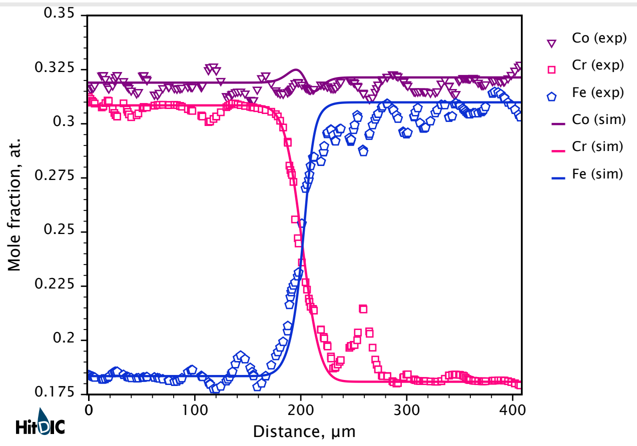

Figure S25 Dabrowa1019-fig13a

1270 K / 345600 sec / 0.0002012394979828968 m

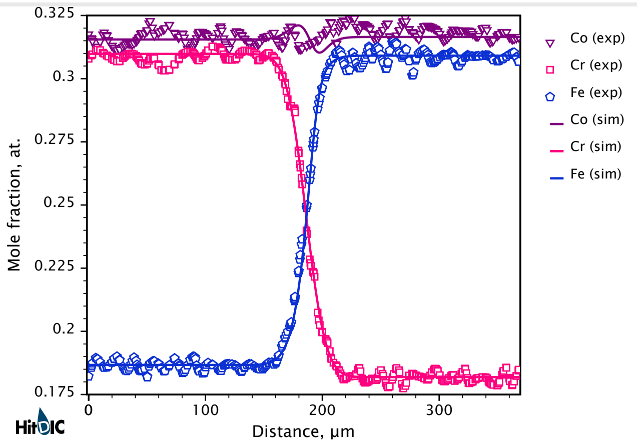

Figure S26 Dabrowa1019-fig13b

1270 K / 345600 sec / 0.000186084202141501 m

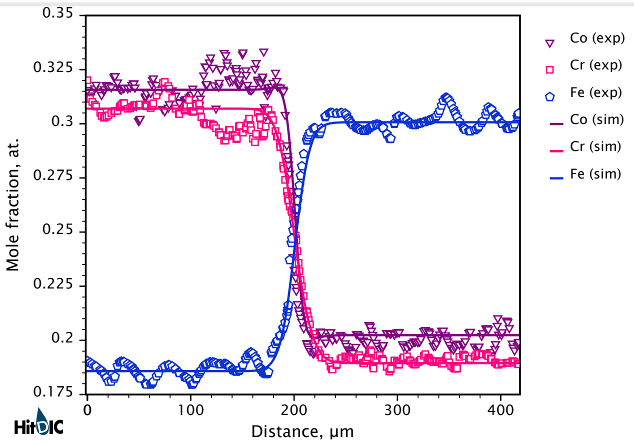

Figure S27 Dabrowa1019-fig13c

1270 K / 345600 sec / 0.00020057789515703917 m

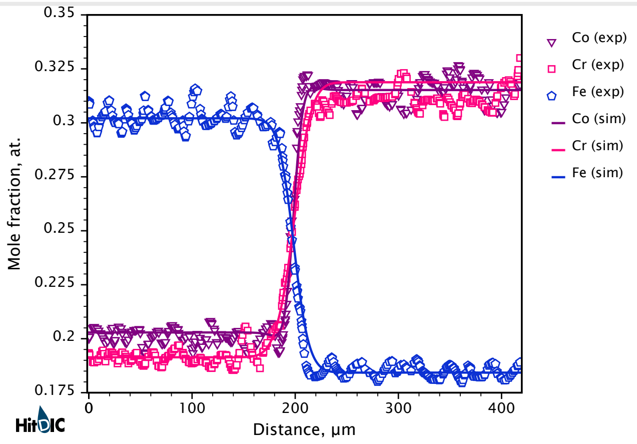

Figure S28 Dabrowa1019-fig13d

1270 K / 345600 sec / 0.0001985968992812559 m

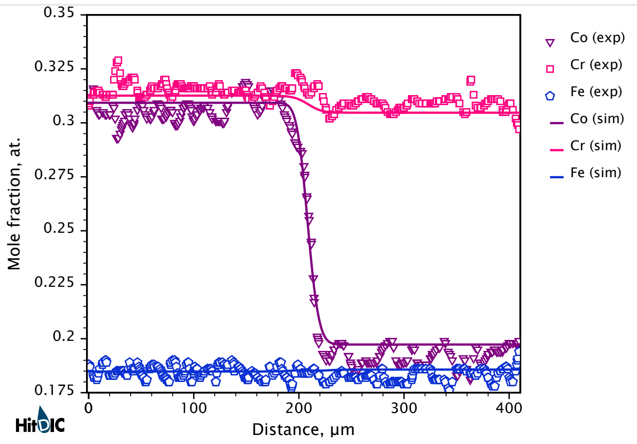

Figure S29 Dabrowa1019-fig13e

1270 K / 345600 sec / 0.00020861449593212456 m

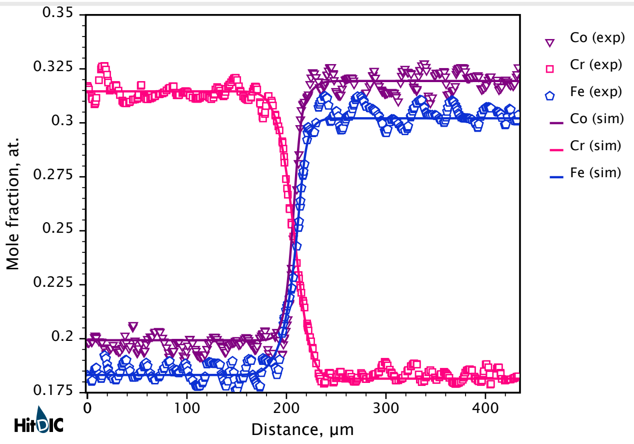

Figure S30 Dabrowa1019-fig13f

1270 K / 345600 sec / 0.0002073711948469281 m

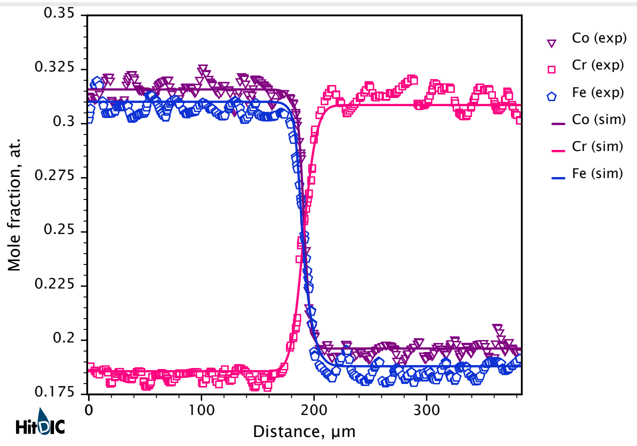

Figure S31 Dabrowa1019-fig13g

1230 K / 345600 sec / 0.0001907276018755510 m

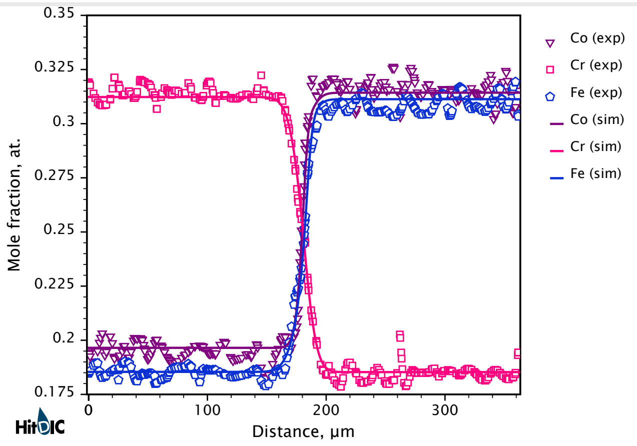

Figure S32 Dabrowa1019-fig13h

1230 K / 345600 sec / 0.0001804688945412635 m

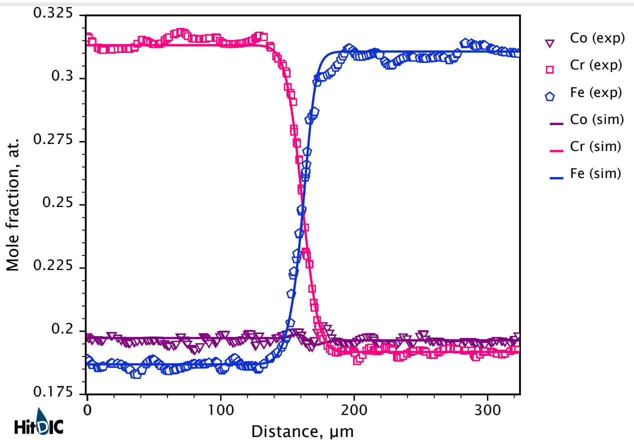

Figure S33 Dabrowa1019-fig14a

1230 K / 345600 sec / 0.0001606891019036993 m

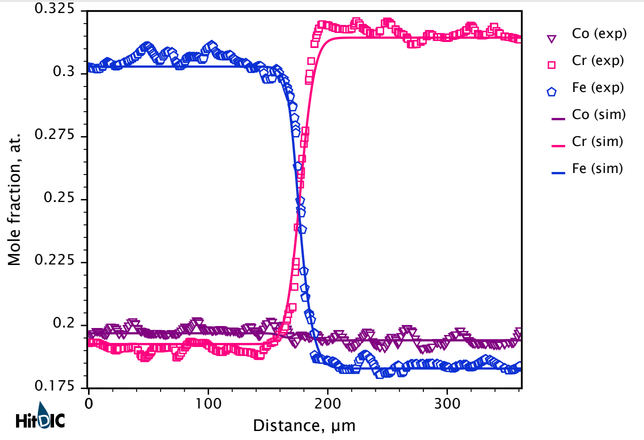

Figure S34 Dabrowa1019-fig14b

1230 K / 345600 sec / 0.00017705329810269177 m

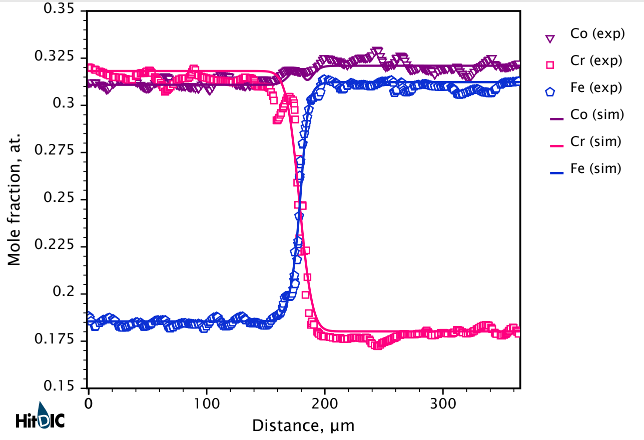

Figure S35 Dabrowa1019-fig14c

1230 K / 345600 sec / 0.0001784449996193871 m

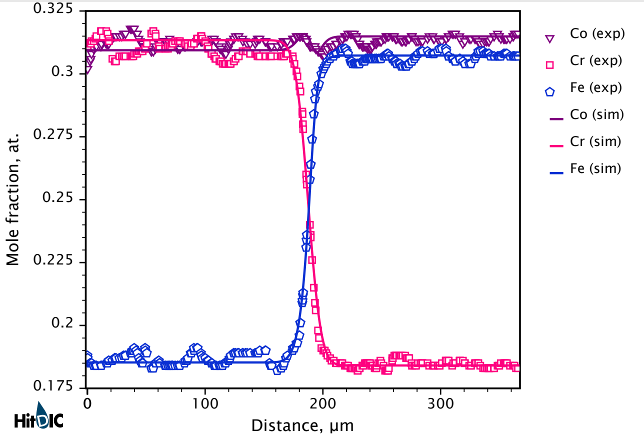

Figure S36 Dabrowa1019-fig14d

1230 K / 345600 sec / 0.00018755860219243914 m

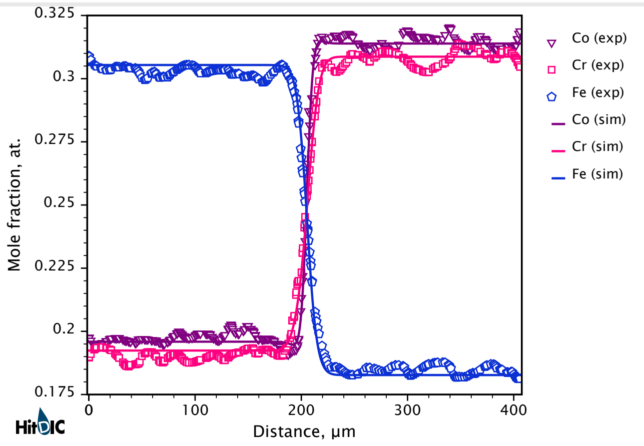

Figure S37 Dabrowa1019-fig14e

1230 K / 345600 sec / 0.0002053775970125571 m

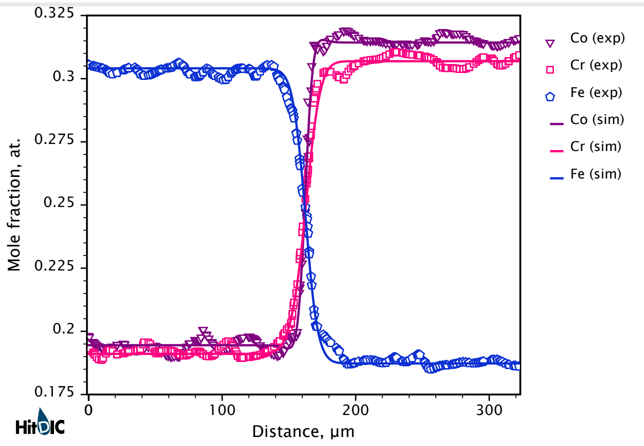

Figure S38 Dabrowa1019-fig14f

1230 K / 345600 sec / 0.0001623448042664677 m

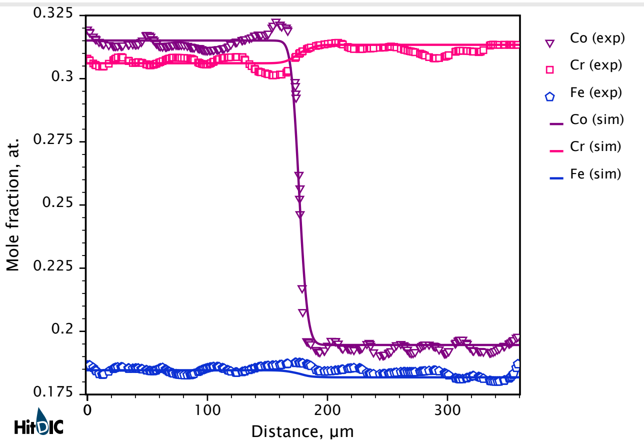

Figure S39 Dabrowa1019-fig14g

1230 K / 345600 sec / 0.00017659149307291955 m

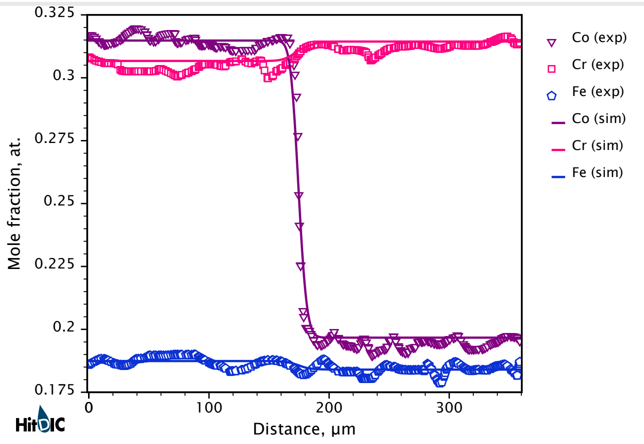

Figure S40 Dabrowa1019-fig14h

1230 K / 345600 sec / 0.0001747053029248491 m

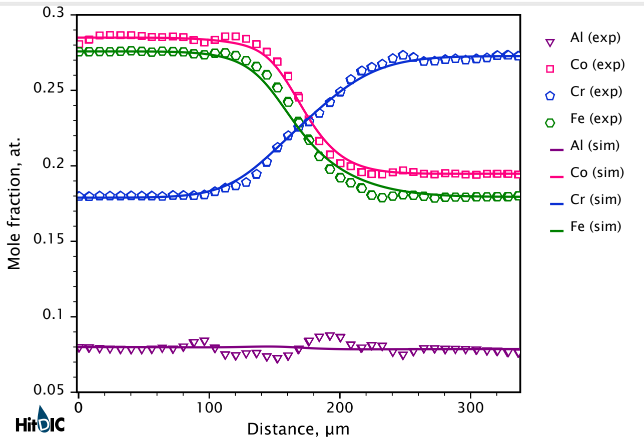

Figure S41 Dabrowa2016-fig10a

1373 K / 180000 sec / 0.00017076499352697283 m

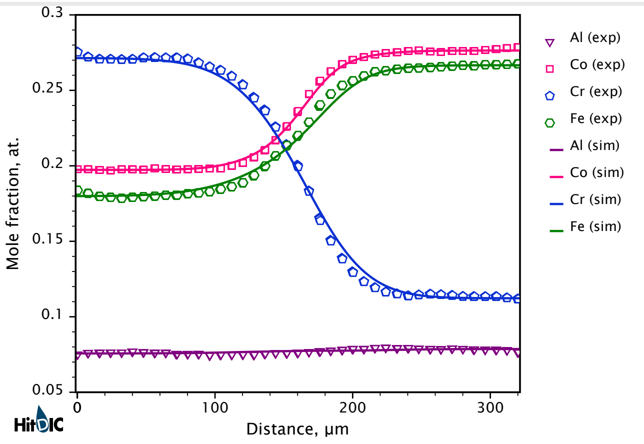

Figure S42 Dabrowa2016-fig10b

1373 K / 180000 sec / 0.00016137599595822394 m

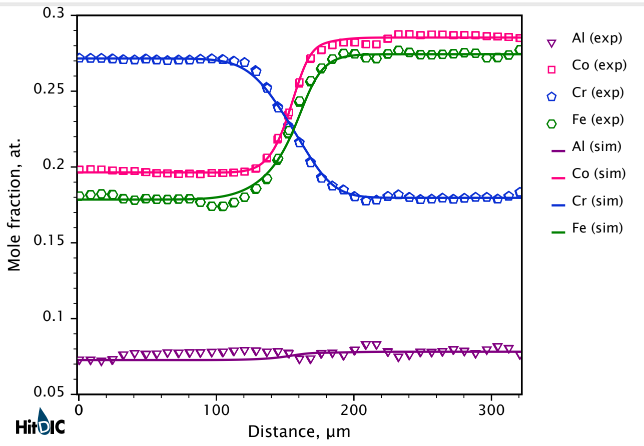

Figure S43 Dabrowa2016-fig6b

1273 K / 360000 sec / 0.00015442799485754222 m

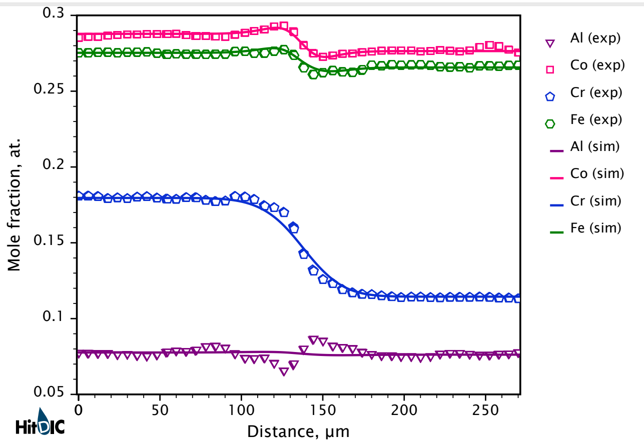

Figure S44 Dabrowa2016-fig7a

1273 K / 360000 sec / 0.00013730100181419402 m

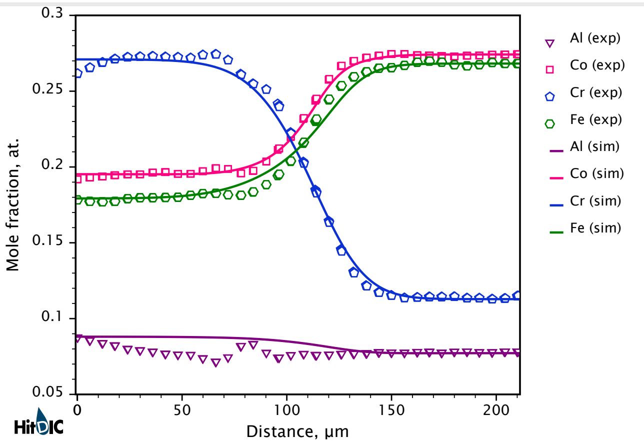

Figure S45 Dabrowa2016-fig7b

1273 K / 360000 sec / 0.00010991800081683323 m

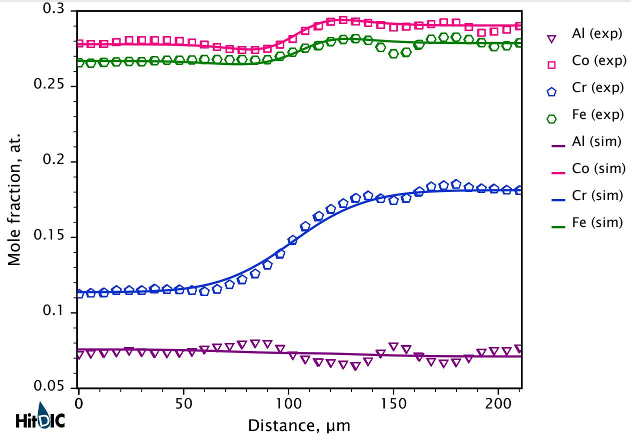

Figure S46 Dabrowa2016-fig8a

1323 K / 270000 sec / 0.00010242900316370651 m

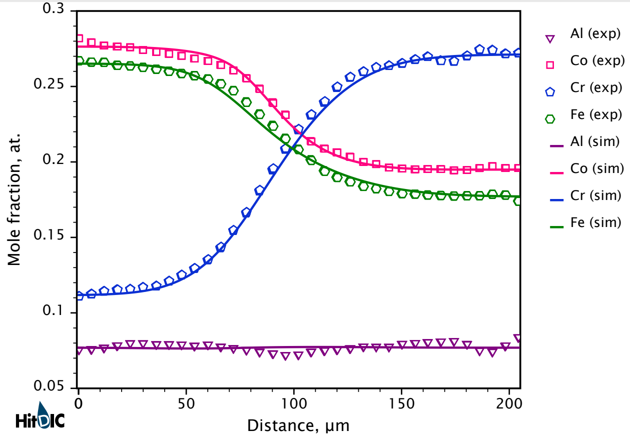

Figure S47 Dabrowa2016-fig8b

1323 K / 270000 sec / 0.00009210669668391347 m

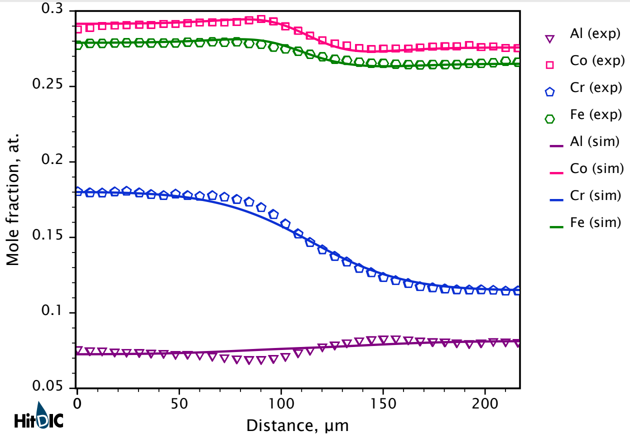

Figure S48 Dabrowa2016-fig9b

1373 K / 180000 sec / 0.00011408999853301793 m

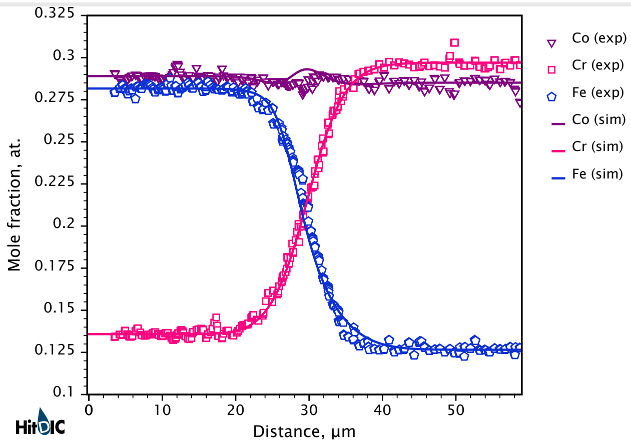

Figure S49 Durand2020-fig6a

1173 K / 360000 sec / 0.00002985520040965639 m

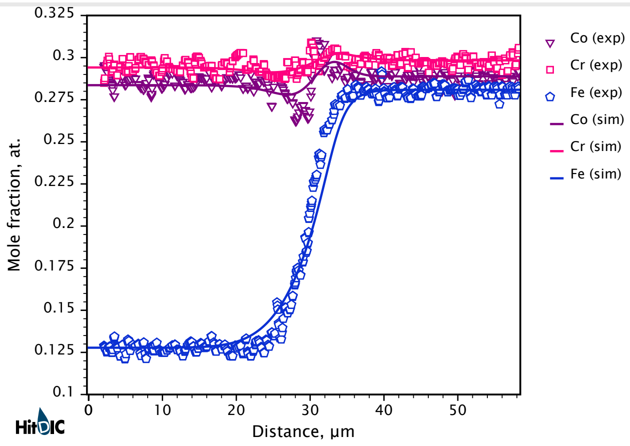

Figure S50 Durand2020-fig6b

1173 K / 360000 sec / 0.000029995100703672506 m

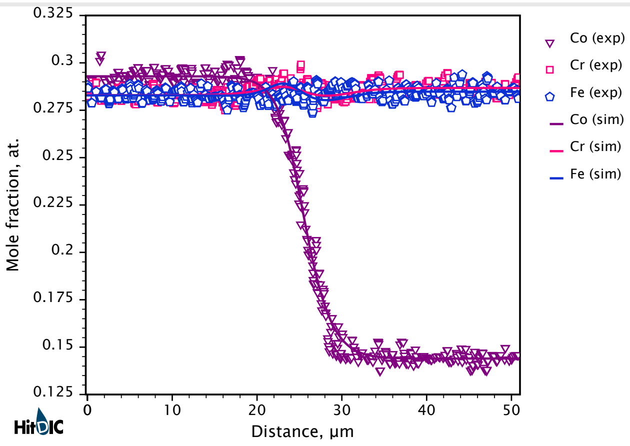

Figure S51 Durand2020-fig6c

1173 K / 360000 sec / 0.000025641900720074773 m

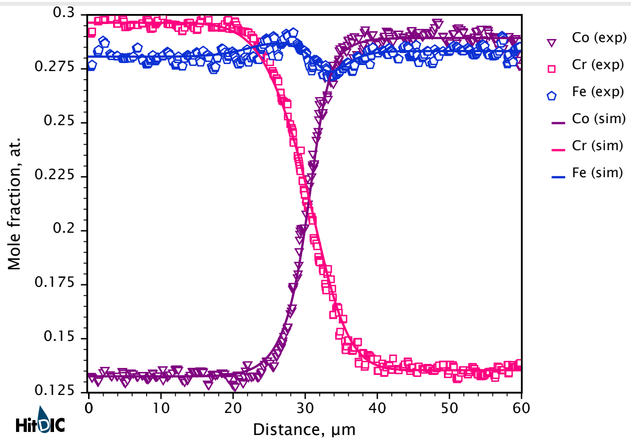

Figure S52 Durand2020-fig6d

1173 K / 360000 sec / 0.000030158200388541445 m

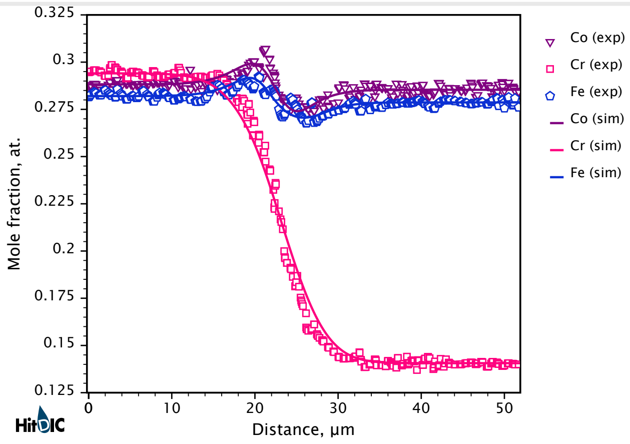

Figure S53 Durand2020-fig6e

1173 K / 360000 sec / 0.00002294389923918061 m

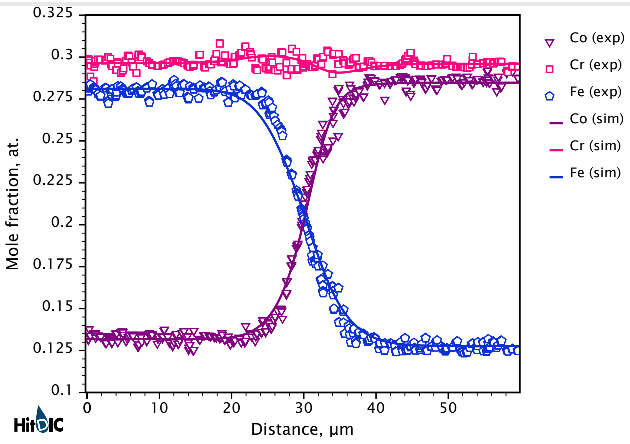

Figure S54 Durand2020-fig6f

1173 K / 360000 sec / 0.000030139199225232005 m

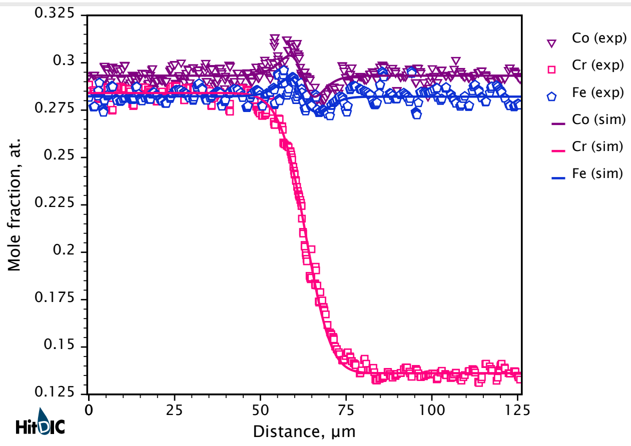

Figure S55 Durand2020-fig7a

1198 K / 360000 sec / 0.00006302599649643525 m

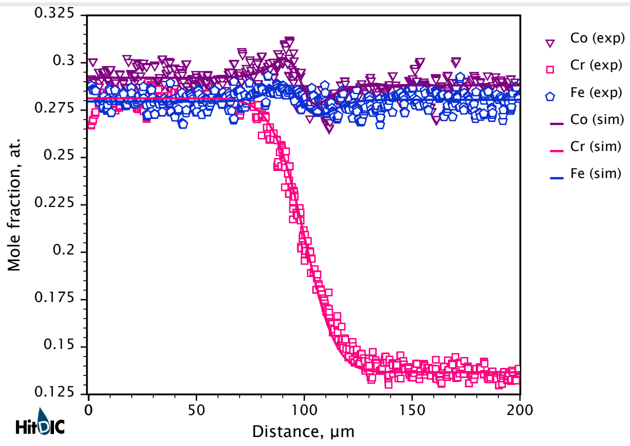

Figure S56 Durand2020-fig7b

1198 K / 1440000 sec / 0.00009964920172933489 m

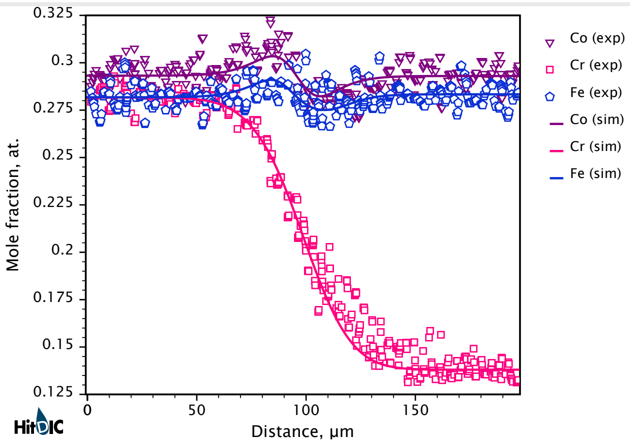

Figure S57 Durand2020-fig7c

1198 K / 3240000 sec / 0.00009780359687283635 m

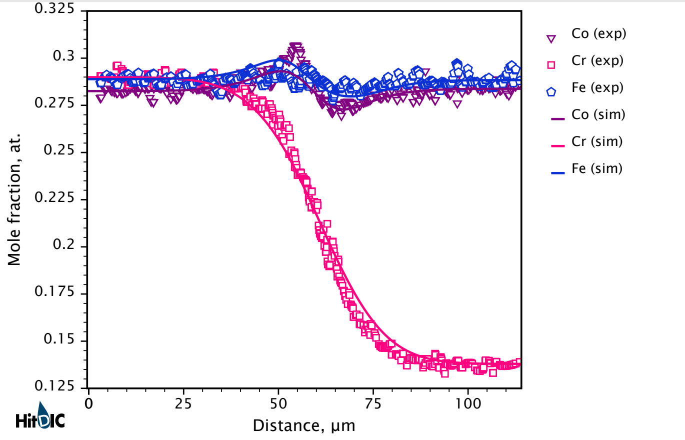

Figure S58 Durand2020-fig7e

1273 K / 360000 sec / 0.000060634098190348595 m

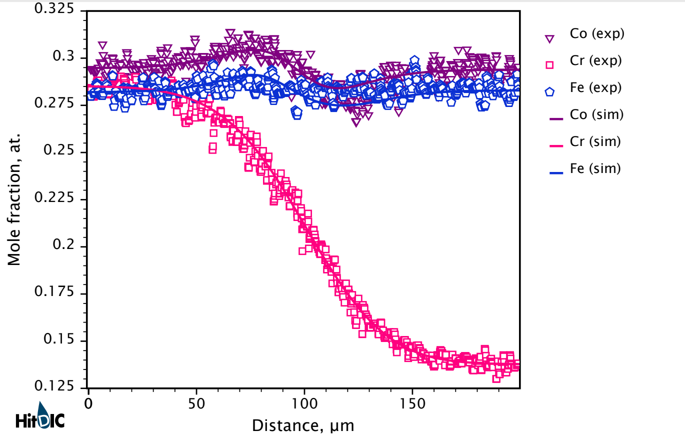

Figure S59 Durand2020-fig7f

1355 K / 360000 sec / 0.00009973740088753402 m

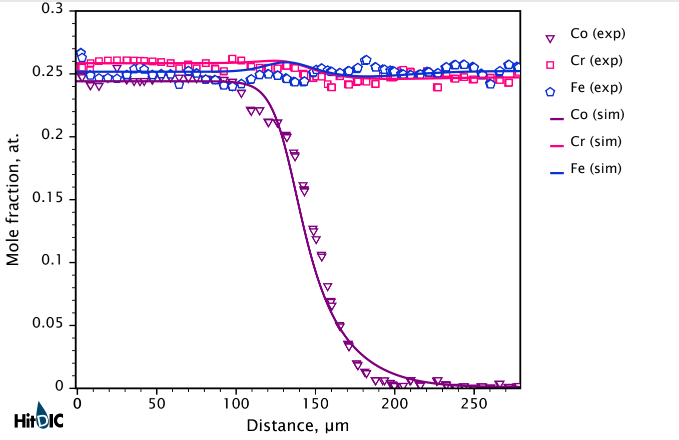

Figure S60 Kejin2018-fig1e

1343 K / 340200 sec / 0.0001484600070398301 m

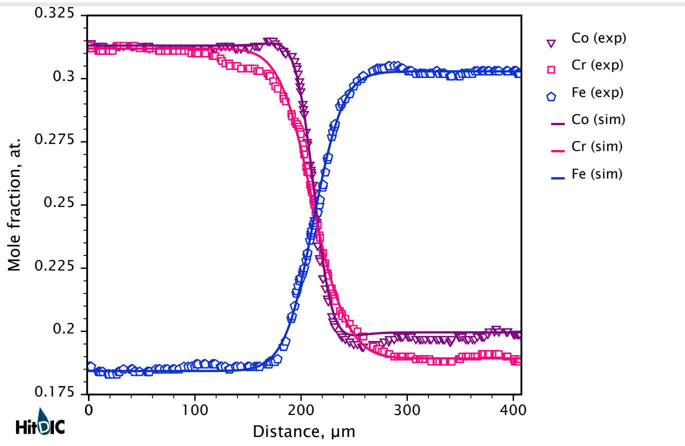

Figure S61 Kucza2018-fig2a

1350 K / 259200 sec / 0.00021175510482862592 m

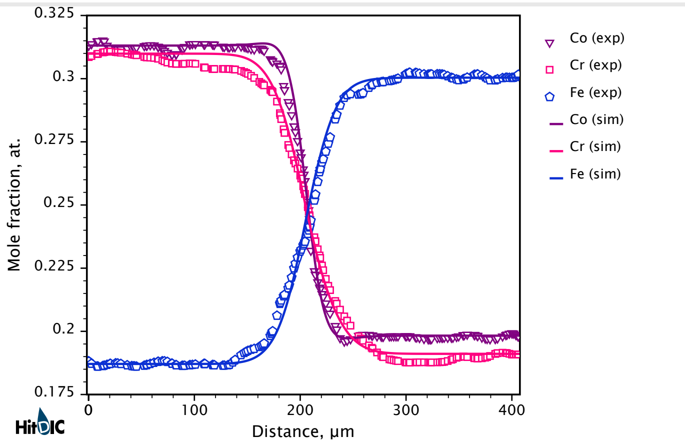

Figure S62 Kucza2018-fig2b

1350 K / 259200 sec / 0.0002054623037111014 m

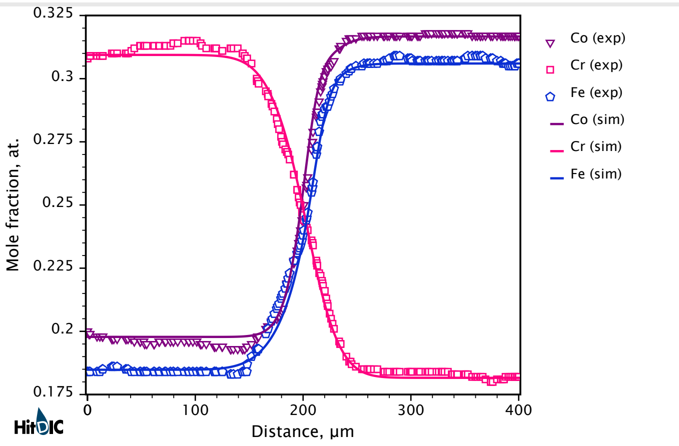

Figure S63 Kucza2018-fig2c

1350 K / 259200 sec / 0.0002007869043154642 m

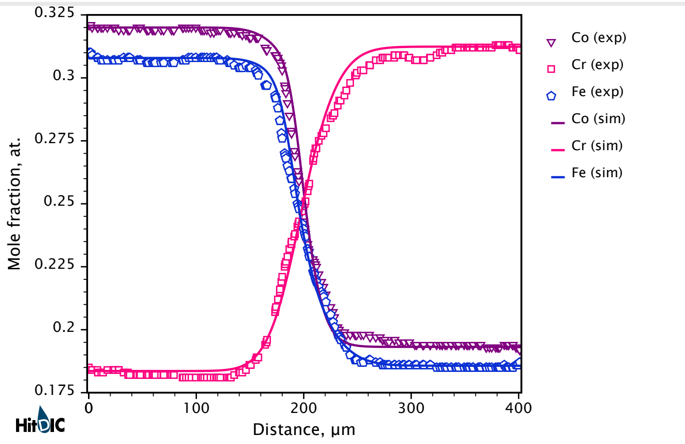

Figure S64 Kucza2018-fig2d

1350 K / 259200 sec / 0.00019931609858758748 μm

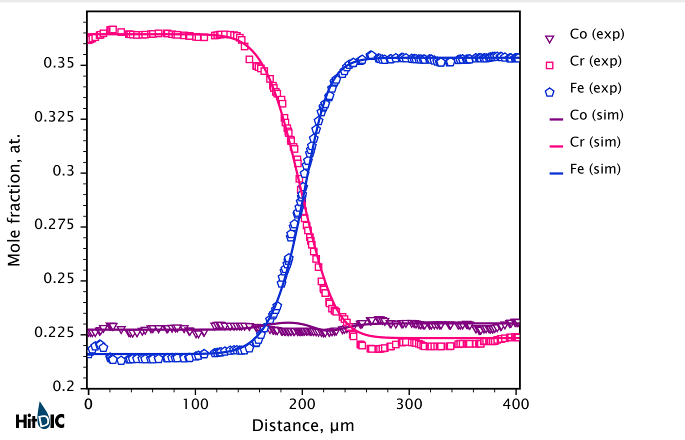

Figure S65 Kucza2018-fig2e

1350 K / 259200 sec / 0.00019747389887925237 μm

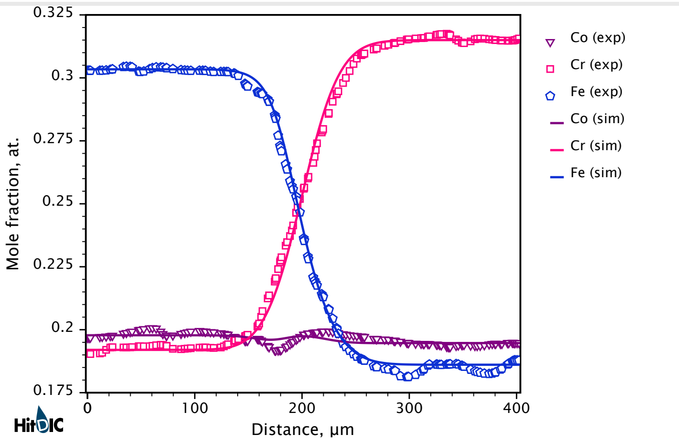

Figure S66 Kucza2018-fig2f

1350 K / 259200 sec / 0.00020033189503010362 μm

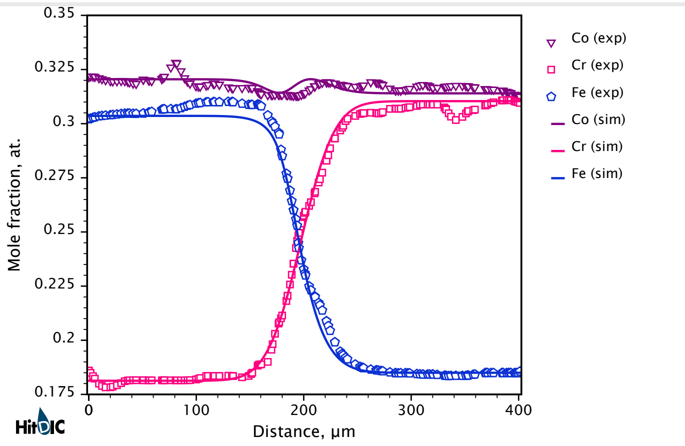

Figure S67 Kucza2018-fig2g

1350 K / 259200 sec / 0.00019733810040634125 μm

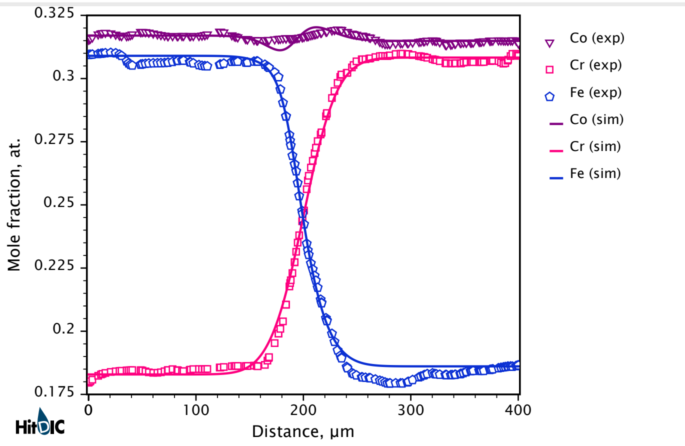

Figure S68 Kucza2018-fig2h

1350 K / 259200 sec / 0.0001998936932068318 μm

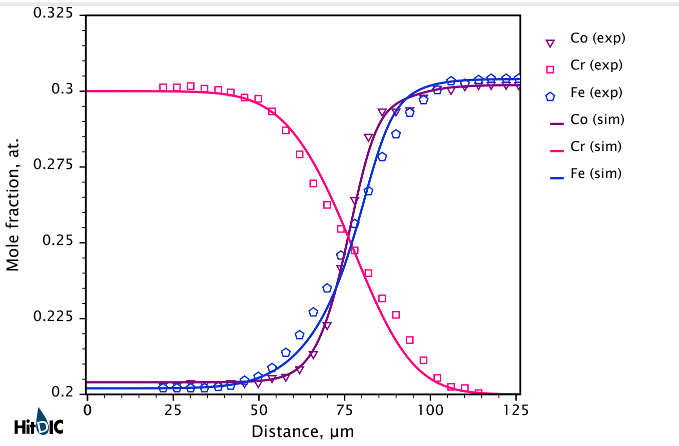

Figure S69 Kulkarni2015

1273 K / 360000 sec / 0.00007634529174538329 μm

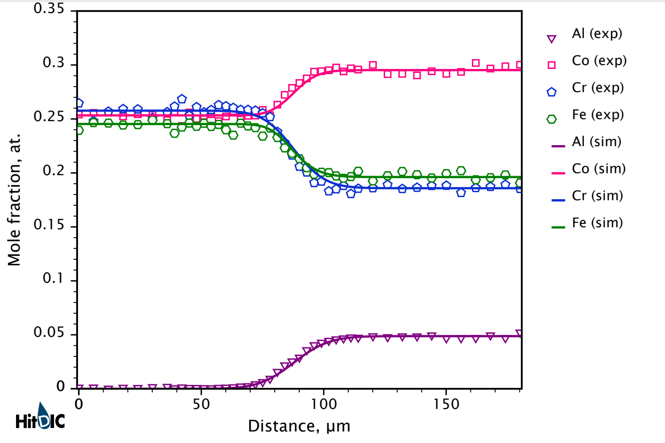

Figure S70 Li2017-1

1273 K / 172800 sec / 0.000088000000068731606 m

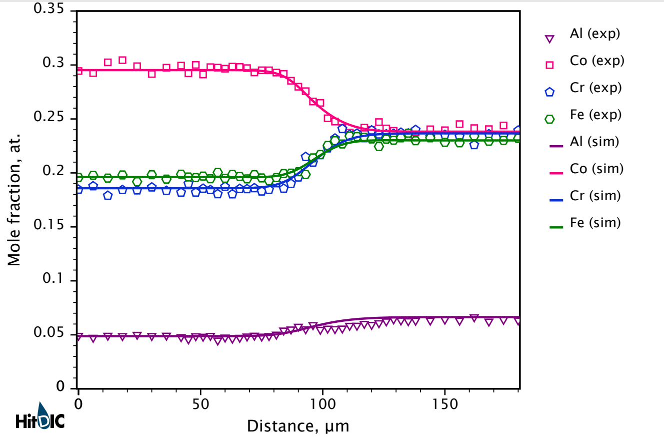

Figure S71 Li2017-2

1273 K / 172800 sec / 0.000096999999645818025 m

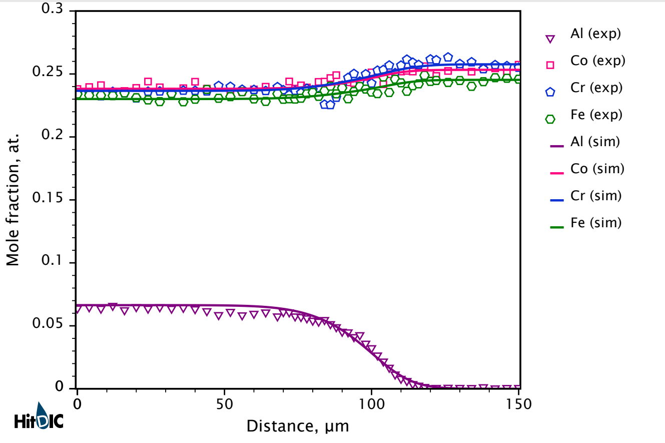

Figure S72 Li2017-3

1273 K / 172800 sec / 0.000096299999840399925 m

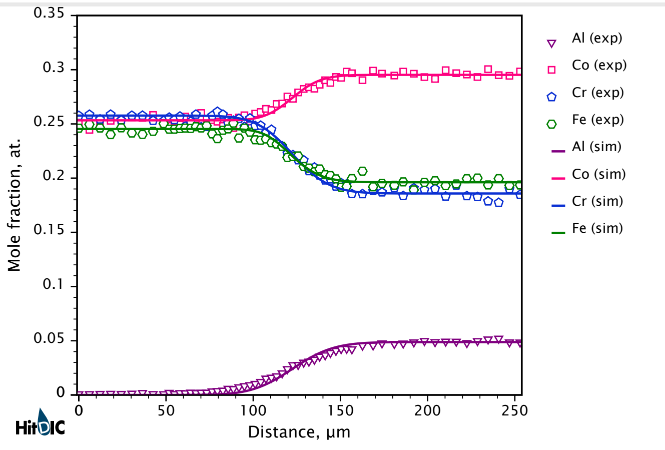

Figure S73 Li2017-4

1323 K / 172800 sec / 0.00012300000052601099 m

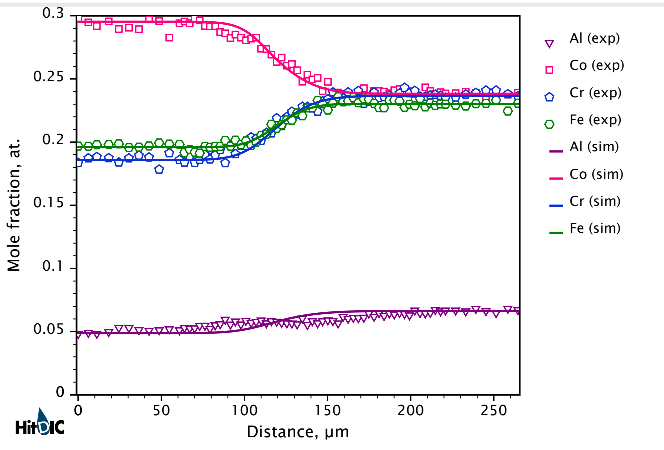

Figure S74 Li2017-5

1323 K / 172800 sec / 0.00011999999696854502 m

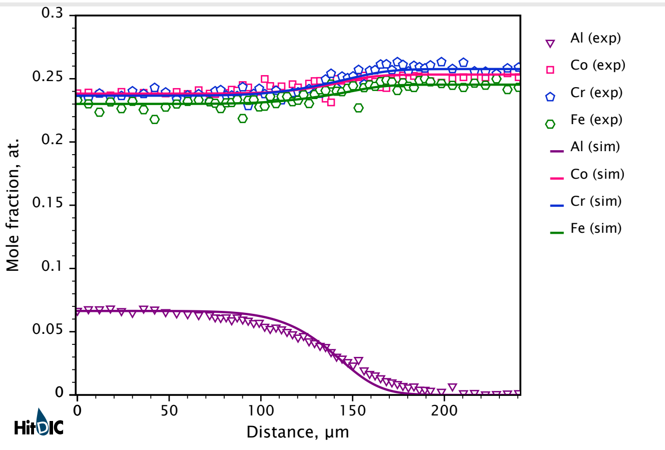

Figure S75 Li2017-6

1323 K / 172800 sec / 0.00013699999544769526 m

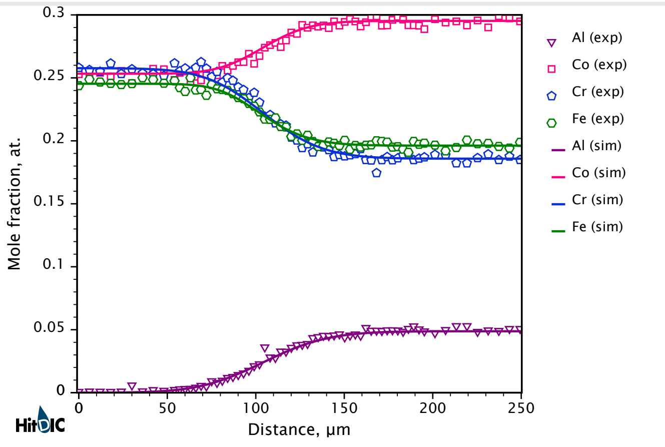

Figure S76 Li2017-7

1373 K / 172800 sec / 0.00010499999991664663 m

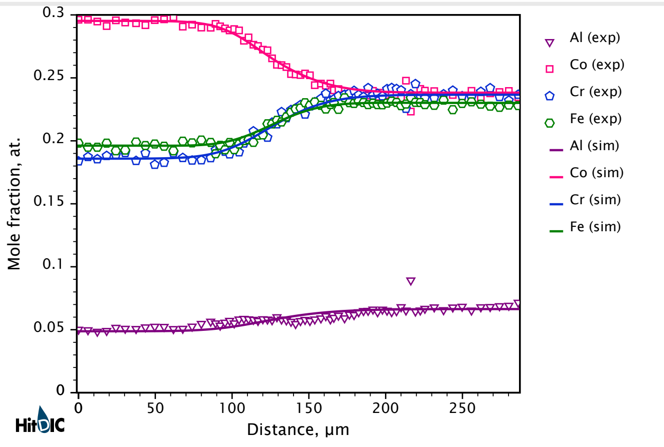

Figure S77 Li2017-8

1373 K / 172800 sec / 0.00012700000661425292 m

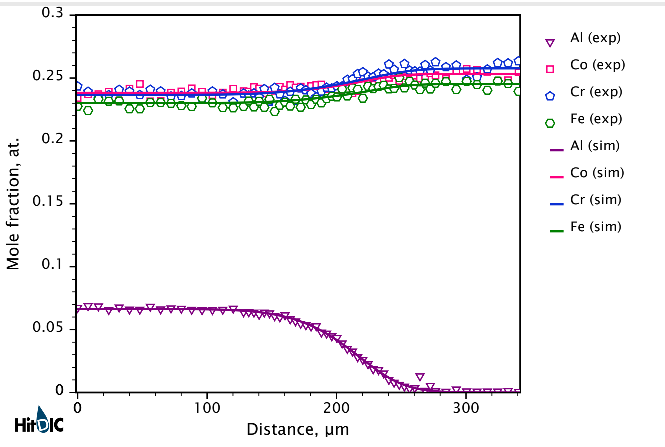

Figure S78 Li2017-9

1373 K / 172800 sec / 0.00020850000146310776 m

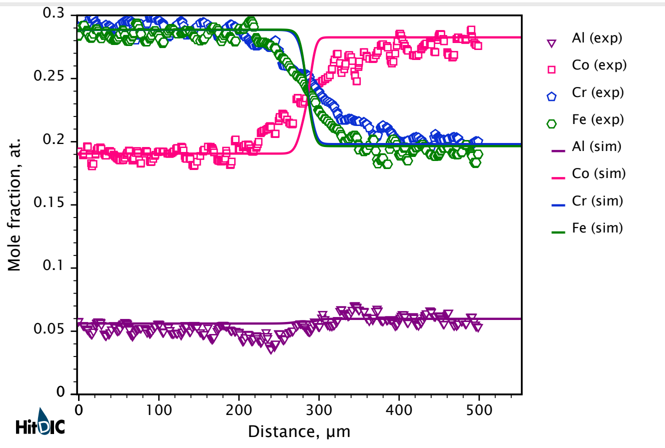

Figure S79 Mehta2021-fig1a

1173 K / 864000 sec / 0.00028499999873433262 μm

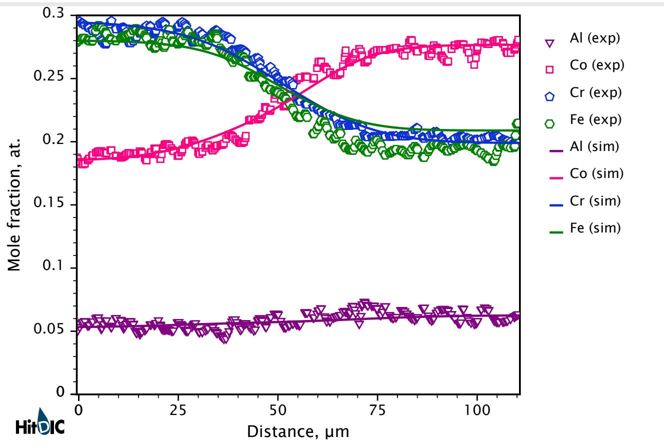

Figure S80 Mehta2021-fig1b

1273 K / 432000 sec / 0.000049999999873689376 μm

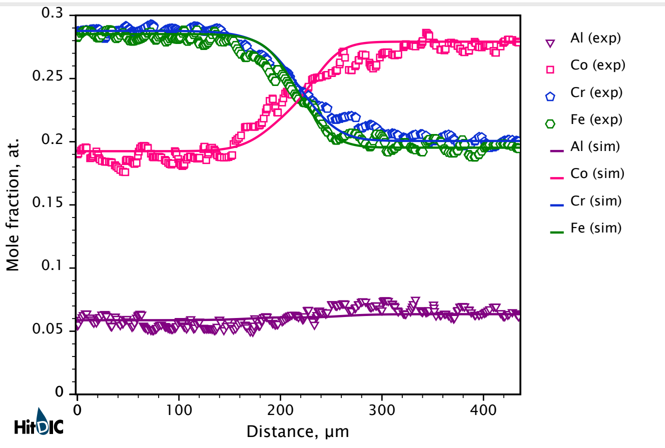

Figure S81 Mehta2021-fig1c

1373 K / 172800 sec / 0.00021699999342672527 μm

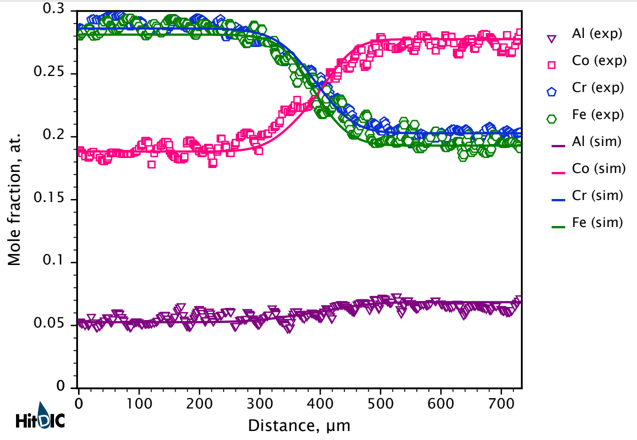

Figure S82 Mehta2021-fig1d

1473 K / 86400 sec / 0.00039000000106170774 m

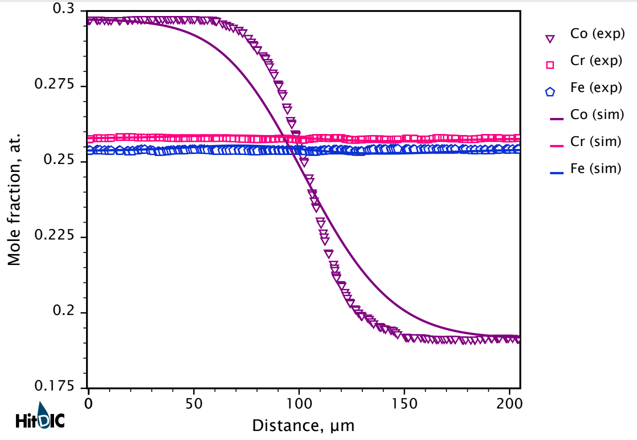

Figure S83 Vaidya2018-fig2a

1423 K / 360000 sec / 0.00010414000280434266 m

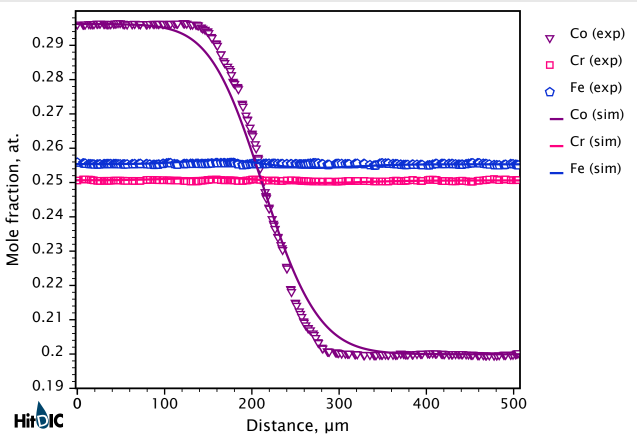

Figure S84 Vaidya2018-fig2b

1423 K / 864000 sec / 0.0002151160006178543 m

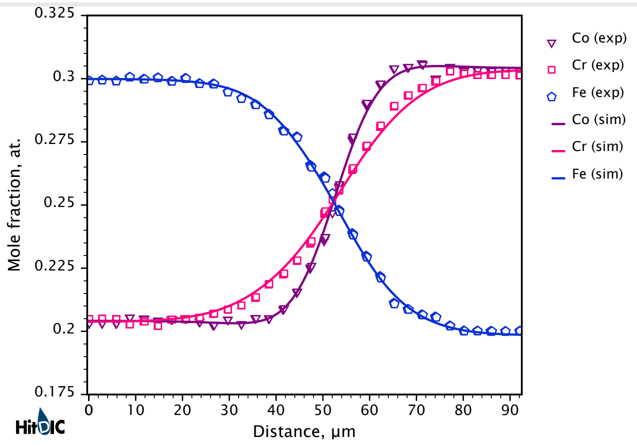

Figure S85 Verma2020-fig2a

1273 K / 360000 sec / 0.00005281850098981522 m

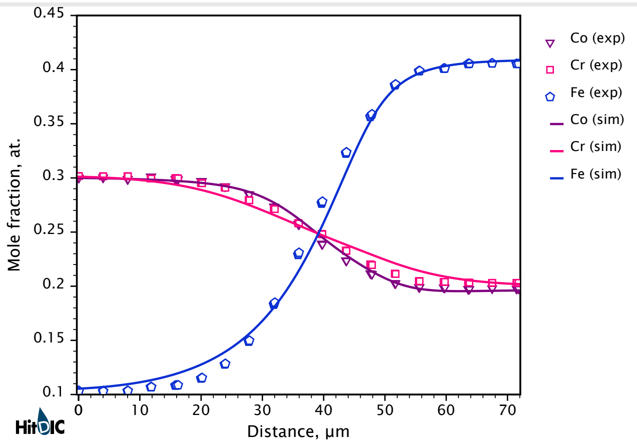

Figure S86 Verma2020-fig2b

1273 K / 360000 sec / 0.00003796159944613464 m

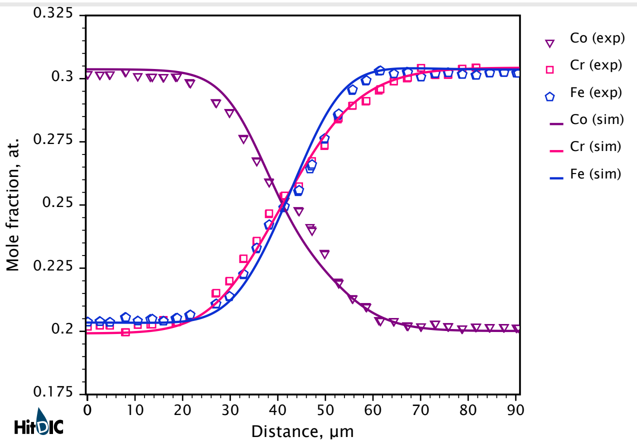

Figure S87 Verma2020-fig2e

1273 K / 360000 sec / 0.0000419313000747934 m

Reference

[Dąbrowa2016] Dąbrowa, J.; Kucza, W.; Cieślak, G.; Kulik, T.; Danielewski, M.; Yeh, J.-W. Interdiffusion in the FCC-Structured Al-Co-Cr-Fe-Ni High Entropy Alloys: Experimental Studies and Numerical Simulations. J. Alloys Compd. 2016, 674, 455–462, doi:10.1016/j.jallcom.2016.03.046.

[Li2017] Li, Q.; Chen, W.; Zhong, J.; Zhang, L.; Chen, Q.; Liu, Z.-K. On Sluggish Diffusion in Fcc Al–Co–Cr–Fe–Ni High-Entropy Alloys: An Experimental and Numerical Study. Metals 2017, 8, 16, doi:10.3390/met8010016

[Mehta2021] Mehta, A.; Sohn, Y. Investigation of Sluggish Diffusion in FCC Al0.25CoCrFeNi High-Entropy Alloy. null 2021, 9, 239–246, doi:10.1080/21663831.2021.1878475.

[Kulkarni2015] Kulkarni, K.; Chauhan, G.P.S. Investigations of Quaternary Interdiffusion in a Constituent System of High Entropy Alloys. AIP Advances 2015, 5, 097162, doi:10.1063/1.4931806.

[Vaidya2018] Vaidya, M.; Mohan Muralikrishna, G.; Divinski, S.V.; Murty, B.S. Experimental Assessment of the Thermodynamic Factor for Diffusion in CoCrFeNi and CoCrFeMnNi High Entropy Alloys. Scripta Mater. 2018, 157, 81–85, doi:10.1016/j.scriptamat.2018.07.040.

[Jinke2018] Jin, K.; Zhang, C.; Zhang, F.; Bei, H. Influence of Compositional Complexity on Interdiffusion in Ni-Containing Concentrated Solid-Solution Alloys. Materials Research Letters 2018, 6, 293–299, doi:10.1080/21663831.2018.1446466.

[Kucza2018] Kucza, W.; Dąbrowa, J.; Cieślak, G.; Berent, K.; Kulik, T.; Danielewski, M. Studies of “Sluggish Diffusion” Effect in Co-Cr-Fe-Mn-Ni, Co-Cr-Fe-Ni and Co-Fe-Mn-Ni High Entropy Alloys; Determination of Tracer Diffusivities by Combinatorial Approach. J. Alloys Compd. 2018, 731, 920–928, doi:10.1016/j.jallcom.2017.10.108.

[Verma2020] Verma, V.; Tripathi, A.; Venkateswaran, T.; Kulkarni, K.N. First Report on Entire Sets of Experimentally Determined Interdiffusion Coefficients in Quaternary and Quinary High-Entropy Alloys. J. Mater. Res. 2020, 35, 162–171, doi:10.1557/jmr.2019.378.

[Dąbrowa2019] Dąbrowa, J.; Zajusz, M.; Kucza, W.; Cieślak, G.; Berent, K.; Czeppe, T.; Kulik, T.; Danielewski, M. Demystifying the Sluggish Diffusion Effect in High Entropy Alloys. J. Alloys Compd. 2019, 783, 193–207, doi:10.1016/j.jallcom.2018.12.300.

[Durand2020] Durand, A.; Peng, L.; Laplanche, G.; Morris, J.R.; George, E.P.; Eggeler, G. Interdiffusion in Cr–Fe–Co–Ni Medium-Entropy Alloys. Intermetallics 2020, 122, 106789, doi:10.1016/j.intermet.2020.106789.
